# Designing gene drives to limit spillover to non-target populations

**DOI:** 10.1371/journal.pgen.1009278

**Published:** 2021-02-25

**Authors:** Gili Greenbaum, Marcus W. Feldman, Noah A. Rosenberg, Jaehee Kim

**Affiliations:** 1 Department of Ecology, Evolution, and Behavior, The Hebrew University of Jerusalem, Jerusalem, Israel; 2 Department of Biology, Stanford University, Stanford, California, United States of America; University of Western Ontario, CANADA

## Abstract

The prospect of utilizing CRISPR-based gene-drive technology for controlling populations has generated much excitement. However, the potential for spillovers of gene-drive alleles from the target population to non-target populations has raised concerns. Here, using mathematical models, we investigate the possibility of limiting spillovers to non-target populations by designing differential-targeting gene drives, in which the expected equilibrium gene-drive allele frequencies are high in the target population but low in the non-target population. We find that achieving differential targeting is possible with certain configurations of gene-drive parameters, but, in most cases, only under relatively low migration rates between populations. Under high migration, differential targeting is possible only in a narrow region of the parameter space. Because fixation of the gene drive in the non-target population could severely disrupt ecosystems, we outline possible ways to avoid this outcome. We apply our model to two potential applications of gene drives—field trials for malaria-vector gene drives and control of invasive species on islands. We discuss theoretical predictions of key requirements for differential targeting and their practical implications.

## Author summary

CRISPR-based gene drive is an emerging genetic engineering technology that enables engineered genetic variants, which are usually designed to be harmful to the organism carrying them, to be spread rapidly in populations. Although this technology is promising for controlling disease vectors and invasive species, there is a considerable risk that a gene drive could unintentionally spillover from the target population, where it was deployed, to non-target populations. We develop mathematical models of gene-drive dynamics that incorporate migration between target and non-target populations to investigate the possibility of effectively applying a gene drive in the target population while limiting its spillover to non-target populations (‘differential targeting’). We observe that the feasibility of differential targeting depends on the gene-drive design specification, as well as on the migration rates between the populations. Even when differential targeting is possible, as migration increases, the possibility for differential targeting disappears. We find that differential targeting can be effective for low migration rates, and that it is sensitive to the design of the gene drive under high migration rates. We suggest that differential targeting could be used, in combination with other mitigation measures, as an additional safeguard to limit gene drive spillovers.

## Introduction

Gene drives are genetic constructs that can bias transmission of desired alleles to progeny, allowing these alleles to rapidly increase in frequency even when they are negatively selected. Gene drives, therefore, have the potential to modify or even eradicate entire species. The population genetics of gene drives, under various genetic architectures, have been studied for several decades [1–9]. With recent innovation in technical engineering of gene drives using CRISPR/Cas9-based methods [10], gene drives have attracted considerable attention for their potential applications. In particular, engineered gene drives can conceivably alter or suppress disease vectors, agricultural pests, or invasive species [11–15].

However, the potential of this technology also raises significant concerns due to the possibility of gene-drive spillovers to non-targeted populations [11, 16–19]. The effects of such spillovers could be devastating, unintentionally driving species to extinction or permanently modifying important traits, potentially leading to ecological cascades [17]. With invasive-species control, there is particular concern, because every invasive species is non-invasive in its native range. Moreover, because an invasion has occurred, it is likely that invaded regions are connected to native ones through migration. As a result, to prevent gene-drive spillovers, every application of a gene drive in an invaded region must be designed to avoid them. For example, it has been suggested that CRISPR-based gene drives could be applied in New Zealand to eradicate invasive species, such as Australian possums, stoats, and rats [15, 17, 19–21]. However, such plans must account for the possibility and potential consequences of spillovers of gene drives from New Zealand to the native ranges of these species. Therefore, understanding the dynamics of gene drives with CRISPR-based constructs under migration, in the context of spillovers to non-target populations, is crucial.

Currently, due to the risks and complications of deploying gene drives, studying spillovers relies on mathematical and computational modeling. CRISPR-based gene-drive models belong to a broader class of preferential-transmission models—systems in which Mendel’s law of equal segregation is violated, with preferential transmission of particular alleles to subsequent generations [22]. Because many general principles of population-genetic theory are violated by non-Mendelian segregation [6, 23], various types of preferential-transmission models have been studied extensively [1–3, 5, 24–28]. Of particular interest are cases in which alleles with enhanced transmission cause reduced fitness of individuals that carry them. Analyses of such models have focused on various types of genetic architectures and evolutionary models [12], such as meiotic drive, modifier genes, and sex-ratio distorters [3, 5, 27, 28].

CRISPR-based gene drives are particular cases of preferential transmission of alleles, and are therefore modeled with the specific features of the CRISPR mechanism. In CRISPR scenarios, the preferential transmission is generated at the zygote formation stage or in the germline by conversion of heterozygotes carrying one copy of the gene-drive allele to homozygotes with two copies of the gene-drive allele [9, 10]. This conversion occurs when the CRISPR mechanism, which is incorporated in the gene-drive allele, edits the other chromosome to replace the wild-type allele with the gene-drive allele.

One of the features of preferential-transmission models is the existence of polymorphic equilibria, namely the states where preferred alleles persist in the population together with wild-type alleles, rather than sweeping to fixation or loss. These equilibria can be either stable or unstable, depending on the genetic architecture involved [1, 2, 4–6, 27, 29]. With CRISPR-based gene-drive technology, it has been suggested that, in a single isolated population, unstable polymorphic equilibria could be utilized for generating a biosafety measure to address the dangers of accidental releases [9]. If a gene drive is initiated at a frequency below an unstable equilibrium, it is expected to be driven to loss, whereas it is expected to be driven towards fixation if initiated at frequencies above the equilibrium [1, 9, 30–32]. Similarly, in spatially continuous populations, it has been argued that a CRISPR-based gene drive with unstable equilibria can be engineered to be driven to fixation only once it is introduced over a sufficiently large area [33].

However, few natural populations exist in isolation, and distinct populations are often connected via migration. Therefore, in order to understand potential consequences of gene-drive spillovers, explicit incorporation of migration between populations into gene-drive models is required. Some studies have examined preferential-transmission migration models with genetic architectures that have not been CRISPR-based [8, 34–39]. Among these studies, some have found that polymorphic equilibria can exist for low migration rates, but not necessarily for high migration rates.

Polymorphic equilibrium states that represent stable conditions under which gene-drive allele frequencies are high in one population but low in another might potentially be exploited to mitigate spillovers. This approach would require (1) identifying stable states in which gene-drive frequencies are high in the target population and low in the non-target population, (2) configuring the genetic architecture of a CRISPR-based gene drive to attain these states, and (3) initiating the gene drive such that it would converge to these states. We term this approach *differential targeting*. In order to consider the prospect of mitigating gene-drive spillovers through differential targeting, gene-drive models that incorporate migration and CRISPR-based genetic architectures are required.

Here, we develop and investigate such models in the context of gene-drive spillovers. We focus on identifying gene-drive designs that allow for differential targeting, and evaluate the feasibility of the approach for mitigating spillovers.

## Results

### Modeling CRISPR-based gene drives with migration

The dynamics of gene drives introduced into a wild population depend in part on features that can, at least in principle, be configured by researchers, such as the gene-drive phenotype and the conversion rate, and in part on features that cannot be controlled, such as the ecological circumstances and life-history traits of the species. In particular, the migration levels between populations are not a controlled feature of the gene drive (unless the gene drive targets dispersal-related traits). Here, in addition to migration, we consider several features of gene drives: (1) the selection coefficients of individuals carrying the gene-drive allele, coefficients that are related to the designed gene-drive phenotype; (2) the life-stage at which the gene-drive phenotype is expressed and subjected to natural selection, specifically whether selection acts before or after the typical migratory life stage or the gene drive conversion; (3) the degree of dominance of the gene-drive allele relative to the wild-type allele; (4) the efficiency of the gene-drive conversion mechanism.

**Fig 1:**
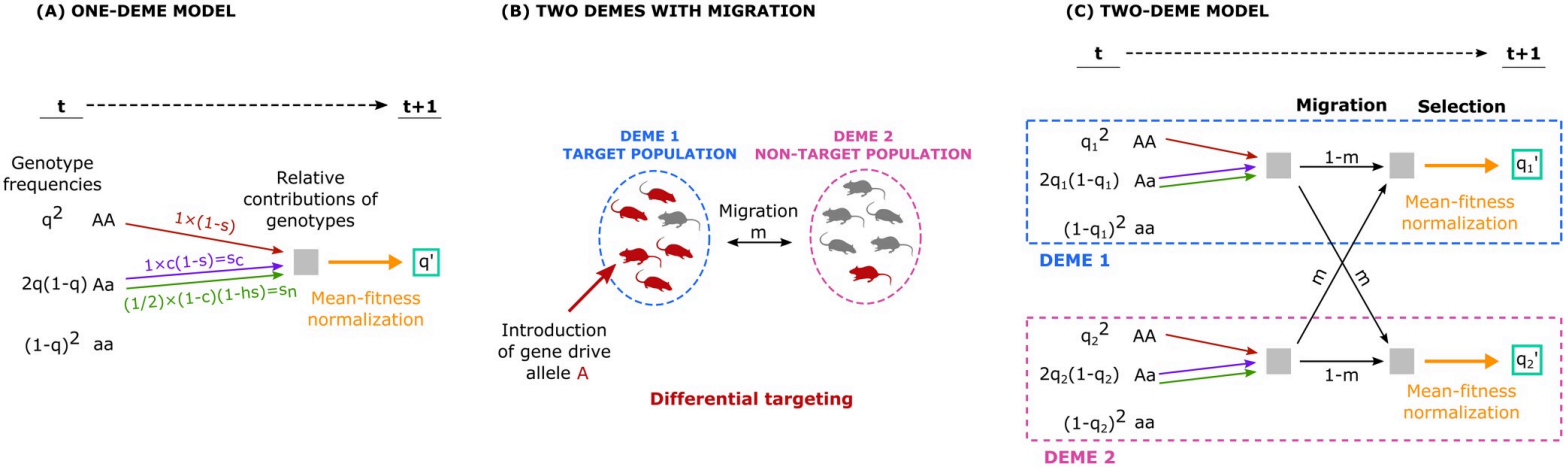
Schematic depiction of gene-drive models. (A) Model of CRISPR-based gene drive in an isolated population (Eq 1). Shown is the change of the allele frequency of the gene-drive allele *A* over one generation, from *t* to *t* + 1. Each genotype contributes to the frequency of *A* in generation *t* + 1 depending on its frequency in generation *t* and the genotype fitnesses: the *AA* genotype contributes an *A* allele (red arrows), and the heterozygous genotype *Aa* contributes 1 (purple arrow) or 1/2 allelic copies (green arrow), depending on whether gene drive conversion occurs (at rate *c*). (B) A two-deme configuration with migration. The gene drive is introduced to the target population, and it can spread to the non-target population through migration. Differential targeting, when possible, would produce convergence to a stable state in which gene-drive frequencies are high in deme 1 and low in deme 2. (C) A two-deme model of gene-drive dynamics in which migration occurs before selection. Black arrows denote migration (Eq 2). The colored arrows (red, green, and purple) represent the contributions of the different genotypes to the next generation’s pre-migration gene pool. $q_{i}^{\prime }$ is calculated by normalizing the relative contributions to the frequency of *A* by the mean fitness of the post-migration population (Eq 3).

We model a CRISPR-based gene drive [10], following previously described models [7, 9]. We consider a population with a wild-type allele, *a*, and a gene-drive allele, *A*, which is initially absent from the population. The gene drive is characterized by (1) the conversion rate *c* of heterozygotes carrying the gene-drive allele to homozygotes of the gene-drive allele (from *Aa* to *AA*), with *c* = 1 being full conversion and *c* = 0 being regular Mendelian inheritance; (2) the degree of dominance *h* of the gene-drive allele; (3) the selection coefficient *s* of a homozygote for the gene-drive allele relative to the wild-type homozygote. In other words, the fitnesses are 1 − *s* for the *AA* genotype, 1 − *hs* for the *Aa* genotype, and 1 for the *aa* genotype. For *s* > 0, the gene-drive allele is negatively selected, and we discuss only this case here, as a beneficial gene drive (*s* < 0) is expected to be driven to fixation in a deterministic model in all connected subpopulations. The evolutionary dynamics of a gene drive can be formulated as a recursion equation describing the change in the frequency of the gene-drive allele, *q*, over one generation [7, 9] (Fig 1A):

$$\begin{array}{c} q^{\prime }=\frac{q^{2}\left(1-s\right)+2q\left(1-q\right)\left(s_{n}+s_{c}\right)}{\bar{w}}. \end{array}$$ (1)

Here, *s*_*n*_ is the contribution to selection of non-converted heterozygotes, *s*_*c*_ is the contribution of converted heterozygotes, and $\bar{w}$ is the mean fitness of the population (see Appendix A in S1 Text for a detailed description of the one-deme model). In this model, $s_{n}=\frac{1}{2}\left(1-c\right)\left(1-hs\right)$, *s*_*c*_ = *c*(1 − *s*) if conversion occurs prior to selection (e.g., conversion in the zygote [10]), and *s*_*c*_ = *c*(1 − *hs*) if conversion occurs after selection [7] (e.g., in the germline [40]); we consider here the case of conversion occurring before selection, and we address conversion after selection in Appendix C in S1 Text.

#### Two-deme model

To incorporate migration between the target population and a non-target population into this modeling framework, we extend the one-deme model in Eq 1. We assume two connected demes, each large and panmictic, with symmetric migration at a rate *m* between them (Fig 1B). As in the one-deme model, the dynamics of the gene drive follow the changes in allele frequencies of the *A* allele in the two demes, *q*_1_ and *q*_2_, respectively. Here, we study a model in which migration occurs before selection, meaning that individuals migrate at a relatively early life stage, and the fitness consequences of the gene-drive phenotypes are expressed in the deme to which the individuals have migrated (e.g., phenotypes expressed at late life stages, such as during reproduction). We have also analyzed two alternative models: selection occurring before migration (Appendix B in S1 Text and S1 Fig), and conversion occurring after migration and selection (Appendix C in S1 Text).

We first consider the allele frequencies of *A* after migration but before selection, ${\tilde{q}}_{1}$ and ${\tilde{q}}_{2}$ in demes 1 and 2, respectively. These frequencies are obtained by accounting for the relative contributions to the *A* allele frequencies of residents and migrants (Fig 1C, black arrows):

$$\begin{array}{c} \begin{array}{c} {\tilde{q}}_{1}=\left(1-m\right)q_{1}+mq_{2}\\ {\tilde{q}}_{2}=\left(1-m\right)q_{2}+mq_{1}. \end{array} \end{array}$$ (2)

Next, we consider the frequencies of *A* after selection takes place on the post-migration gene pools (Fig 1C, orange arrows). The changes in the allele frequencies between generations in each population are obtained as in Eq 1, using the post-migration frequencies from Eq 2:

$$\begin{array}{c} \begin{array}{c} q_{1}^{\prime }=\frac{{\tilde{q}}_{1}^{2}\left(1-s\right)+2{\tilde{q}}_{1}\left(1-{\tilde{q}}_{1}\right)\left(s_{n}+s_{c}\right)}{{\bar{w}}_{1}}\\ q_{2}^{\prime }=\frac{{\tilde{q}}_{2}^{2}\left(1-s\right)+2{\tilde{q}}_{2}\left(1-{\tilde{q}}_{2}\right)\left(s_{n}+s_{c}\right)}{{\bar{w}}_{2}}, \end{array} \end{array}$$ (3)

where ${\bar{w}}_{1}$ and ${\bar{w}}_{2}$ are the post-migration mean fitnesses of demes 1 and 2, respectively, expressed as ${\bar{w}}_{i}={\tilde{q}}_{i}^{2}\left(1-s\right)+2{\tilde{q}}_{i}\left(1-{\tilde{q}}_{i}\right)\left(2s_{n}+s_{c}\right)+{\left(1-{\tilde{q}}_{i}\right)}^{2}$.

### Differential-targeting equilibria (DTEs)

In order to understand the evolutionary trajectories of the gene drive in the two-deme system, we study the equilibrium states of the model. This is accomplished by solving Eq 3 under the equilibrium conditions $q_{1}^{\prime }=q_{1}$ and $q_{2}^{\prime }=q_{2}$. We denote these solutions, the equilibrium points in frequency space, by $\left({\hat{q}}_{1},{\hat{q}}_{2}\right)$, where ${\hat{q}}_{1}$ and ${\hat{q}}_{2}$ are the equilibrium frequencies in demes 1 and 2, respectively. We consider only solutions for which both ${\hat{q}}_{1}$ and ${\hat{q}}_{2}$ lie in the interval [0, 1], and we analyze the stability of these equilibria (see Methods).

A gene-drive configuration is denoted (*s*, *c*, *h*), where *s*, *c*, and *h* can have any value in the interval [0, 1]. Under any gene-drive configuration, there are two trivial equilibrium points, corresponding to global fixation, $\left({\hat{q}}_{1},{\hat{q}}_{2}\right)=\left(1,1\right)$, and global loss, $\left({\hat{q}}_{1},{\hat{q}}_{2}\right)=\left(0,0\right)$, of the gene-drive allele. In the one-deme model, it has been demonstrated that for some gene-drive configurations (*s*, *c*, *h*), there exists a single non-trivial (polymorphic) equilibrium point, which could be either stable or unstable [7, 9]. For these configurations, the stabilities of the equilibria are alternating, i.e., the two trivial equilibria are stable and the non-trivial equilibrium is unstable, or the two trivial equilibria are unstable and the non-trivial equilibrium is stable.

In order to understand how different gene-drive configurations result in different equilibrium states, we partition the set of possible configurations of (*s*, *c*, *h*) into four subsets (S3–S9 Figs) based on their behaviors in the one-deme model (Eq 1). The first two subsets consist of those configurations for which there exist only the two trivial equilibria, one of which is stable: (1) *A*_1_, the set of configurations for which the gene-drive allele eventually reaches fixation, and (2) *A*_2_, the set of configurations for which the gene-drive allele is eventually lost. The other two subsets are those for which there is a non-trivial equilibrium, in addition to the two trivial ones: (3) *B*_1_, the set of configurations for which the additional non-trivial equilibrium is stable, and (4) *B*_2_, the set of configurations for which the additional non-trivial equilibrium is unstable. The *B*_2_ configurations are of particular interest, because they represent threshold-dependent gene drives, which spread only if initiated at frequency above the unstable equilibrium [7, 9].

**Fig 2:**
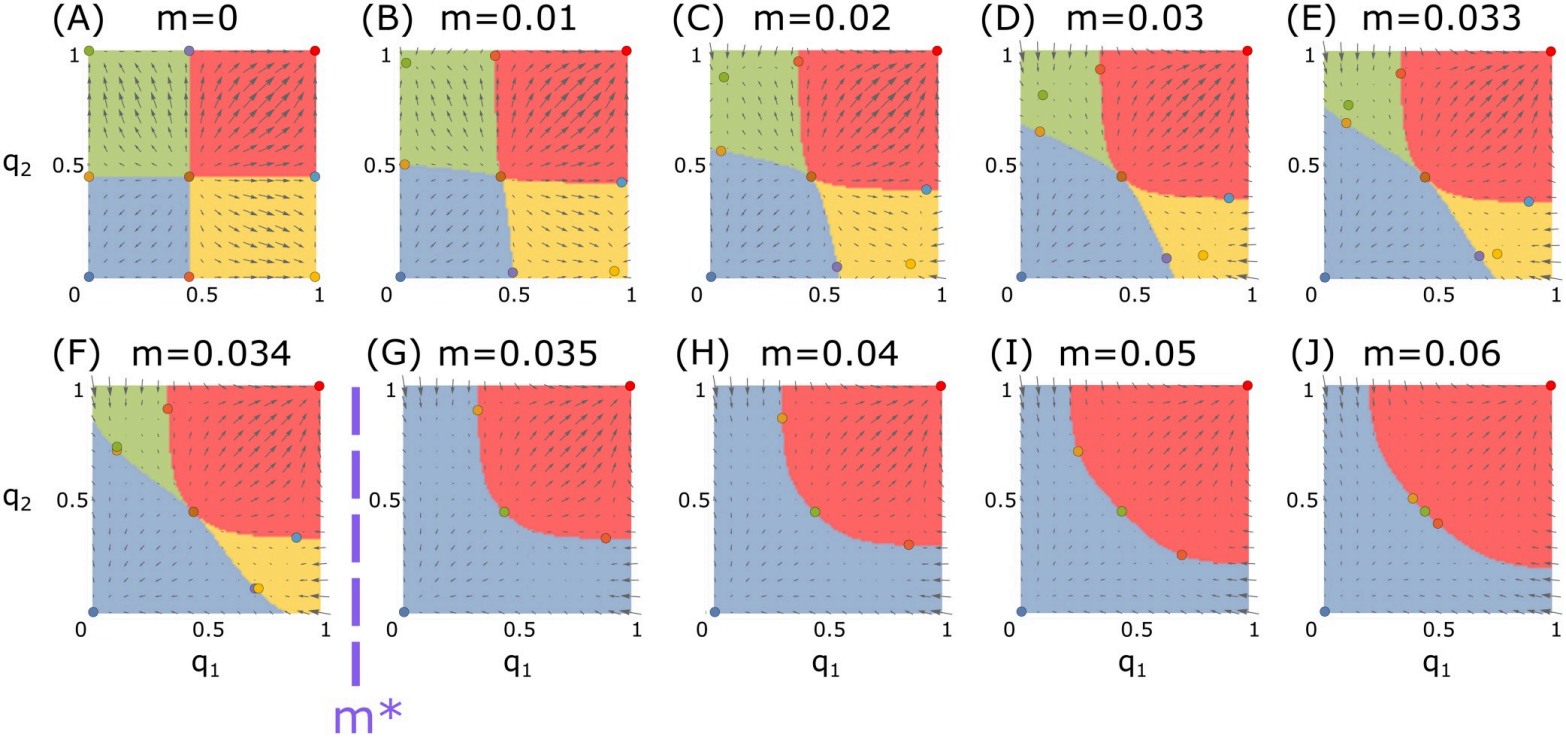
Equilibria and basins of attractions for different migration rates. Shown are results with a gene-drive configuration (*s*, *c*, *h*) of (0.6, 0.8, 0) (a configuration in *B*_2_). The circles show the equilibria. The colored regions show the attraction basins, with the basin colors corresponding to the stable equilibria. The arrows show the vector field that describes the magnitude and direction of the change in allele frequencies at each point in frequency space. The differential-targeting equilibrium (DTE) is the stable yellow equilibrium point, which exists for migration rates lower than m* ≈ 0.023.

We can leverage the results obtained for the one-deme model to explore equilibria in the two-deme model. For the two-deme model, the number of equilibrium solutions for Eq 3 depends on the gene-drive configurations (*s*, *c*, *h*), and also on the migration rate *m* (Fig 2). For tractability, we first consider an ecologically uninteresting yet illustrative case, where *m* = 0. In this case of no migration, the two-deme system becomes two independent one-deme systems. For *B*_1_ or *B*_2_ configurations, the number of equilibria in each one-deme system is 3, and therefore, the number of equilibria in the two disconnected demes is 3 × 3 = 9 (for example, Fig 2A). Of these equilibria, one equilibrium point, (1, 0), represents a desired outcome, in which the target deme (population 1) is affected by the gene drive, which is absent from the non-target deme (population 2). This equilibrium is stable only for *B*_2_ configurations, in each of the one-deme systems and hence in the two-deme system, and it is unstable for *B*_1_ configurations. Therefore, if a gene drive with a configuration in *B*_2_ is initiated in the basin of attraction of the stable equilibrium point (1, 0) (e.g., yellow region in Fig 2A), then we expect that the desired outcome—the gene drive sweeping the target population and not the non-target population—will be reached.

Our main interest is to understand whether similar desired outcomes, in which a stable equilibrium exists with high gene-drive frequencies in the target population and low frequencies in the non-target population, can exist for *m* > 0. We term such an equilibrium point $\left({\hat{q}}_{1},{\hat{q}}_{2}\right)$, for which ${\hat{q}}_{1}>{\hat{q}}_{2}$, a *differential-targeting equilibrium* (DTE). In other words, we define an equilibrium point as a DTE if it is stable, and if the equilibrium gene-drive frequency in the target deme is larger than the frequency in the non-target deme. If a DTE exists, and we initiate the gene drive in its basin of attraction, then the system converges to frequencies that are higher in the target population than in the non-target population.

For *m* > 0, we obtain the possible number and type of equilibria attainable for Eq 3 by numerically exploring the parameter space of possible gene-drive configurations and migration rates. In previous work for the specific case of *m* = 0, it has been seen that if (1, 0) or (0, 1) are stable equilibria, then stable non-trivial equilibria will exist if *m* > 0 is small enough [34, 35]. Although the gene-drive configuration sets *A*_1_, *A*_2_, *B*_1_, and *B*_2_ were defined for the one-deme model rather than for the two-deme model, and they are independent of *m*, they play an important role for investigating the existence of DTEs. For *B*_2_ configurations, and only for *B*_2_ configurations, we numerically find that 9 equilibria exist for some *m* > 0. Moreover, 9 equilibria exist only for low *m* > 0, whereas for higher migration rates there exist fewer equilibria (S4–S9 Figs). For example, 9 equilibria exist in Fig 2A–2F, but only 5 exist in Fig 2G–2J. Only when 9 equilibria exist does there exist a DTE, and this DTE is always unique. For example, in Fig 2A–2F, the yellow points are DTEs, and the yellow regions around them are the corresponding basins of attraction. At the DTE, the gene drive is maintained at a considerably lower frequency in the non-target population than in the target population.

Notably, we observe that DTEs exist only for low migration rates (S4–S9 Figs). For *B*_2_ configurations, when migration exceeds a certain critical threshold, there are no longer 9 equilibrium solutions to Eq 3, but only 5 or 3 solutions, none of which are both stable and allow differential targeting (S4–S9 Figs). We label this critical threshold of existence of a DTE by m*(*s*, *c*, *h*) (Fig 2). In other words, m*(*s*, *c*, *h*) is defined as the supremum of the set of migration rates *m* for which there are 9 equilibria for Eq 3 with the parameters *s*, *c*, *h*, and *m*. This set of migration rates is not empty, because for *m* = 0 there are 9 equilibria for *B*_2_ configurations, as shown above, and therefore the supremum m* is well-defined. For *m* > m*, we observe that all stable equilibria are symmetric (${\hat{q}}_{1}={\hat{q}}_{2}$). For (*s*, *c*, *h*) configurations that are not in *B*_2_, m* is undefined, because in these cases no DTEs exist for any *m* > 0.

We investigated m* numerically across the parameter space. We observe that for scenarios where *m* > m*, only two stable equilibria exist—global fixation (1, 1) and global loss (0, 0). Hence, differential-targeting of a gene drive is not possible if *m* > m*, and spillover to deme 2 of a gene drive that affects deme 1 is unavoidable. For *A*_1_, *A*_2_, and *B*_1_ configurations and for any *m* > 0, the results in the two-deme models are equivalent to those in the one-deme model. In other words, for each equilibrium ${\hat{q}}_{i}$ in the one-deme model, $\left({\hat{q}}_{i},{\hat{q}}_{i}\right)$ is the corresponding equilibrium in the two-deme model, with the same stability properties.

### Migration and differential targeting

**Fig 3:**
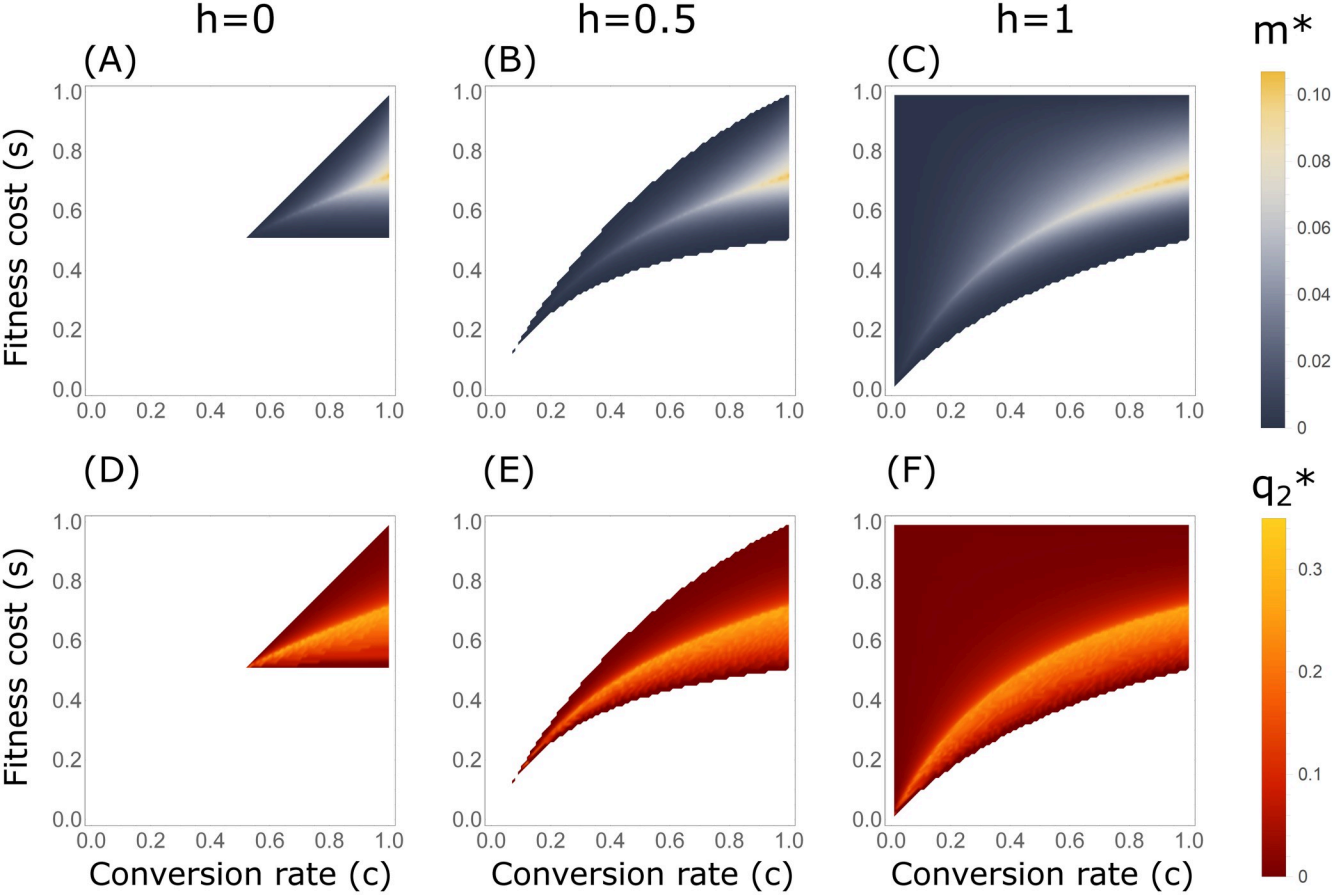
Critical migration thresholds for differential-targeting in the two-deme model. (A–C) Maximal migration rates m* for which a DTE exists. The colored regions denote the configurations for which a DTE exists (*B*_2_ configurations), and the regions in white denote configurations for which differential targeting of the demes is not possible (*A*_1_, *A*_2_, and *B*_1_). Over most of the parameter space, a DTE exists only for low migration rates (in blue), and only in a narrow band do DTEs exist with migration rates above m* = 0.05 (light yellow). (D–F) Maximal gene-drive frequencies in the non-target population, $q_{2}^{*}$, at DTEs. $q_{2}^{*}$ values are correlated with m* values.

We obtained m* numerically for all configurations (*s*, *c*, *h*) in *B*_2_ using the numerical equilibrium solution for Eq 3 (Fig 3A–3C). We find that for most of the parameter range, m* is low (blue regions in Fig 3A–3C), except in a narrow curved band across the parameter space (pale yellow bands in Fig 3A–3C). m* increases for higher conversion rates *c*, and is maximal (m* ≈ 0.110) for *c* = 1 and *s* ≈ 0.72; we denote this maximizing selection coefficient by s*. Note that *h* values are not relevant for a full-conversion gene drive (*c* = 1), because in such configurations, the system has no heterozygous individuals.

#### Impact of differential targeting on the non-target population

In practice, as the migration rate *m* cannot be estimated accurately and may vary over time, it is important to understand the potential consequences for the non-target population of differential targeting, for a range of values of *m*. In principle, assuming the goal is suppression of the population in deme 1, initiation of the gene drive in the basin of attraction of the DTE leads to a high frequency of *A* in deme 1 and a low frequency in deme 2. This state is maintained until the population in deme 1 begins to collapse due to the population-level impact of the gene drive. For these generations, the population in deme 2, the non-target population, experiences the burden of the gene drive at frequency ${\hat{q}}_{2}$ of the DTE.

For a given configuration (*s*, *c*, *h*) in *B*_2_, we define the supremum equilibrium frequency of the DTE in the non-target population for migration rates below m* as $q_{2}^{*}\left(s,c,h\right)=\underset{m<m*}{\sup}{\hat{q}}_{2}\left(s,c,h,m\right)$, where ${\hat{q}}_{2}\left(s,c,h,m\right)$ is the gene-drive frequency at the DTE with configuration (*s*, *c*, *h*) under migration rate *m*. We computed $q_{2}^{*}$ numerically across the parameter range *B*_2_ (Fig 3D–3F). In general, we find that $q_{2}^{*}$ is positively correlated with m* (Fig 3), meaning that gene-drive configurations that can sustain differential targeting for higher migration rates also potentially result in higher frequencies of the gene drives in the non-target population. Considering all possible gene-drive configurations in *B*_2_, $q_{2}^{*}$ is maximal for *c* = 1 and *s* = s*, the same configuration that maximizes m*. This maximal allele frequency of *A* in the non-target population at a DTE is $q_{2}^{*}=0.28$. The configurations that produce these $q_{2}^{*}$ values, with *c* = 1 and *s* = s*, delineate the conditions at which differential targeting influences the non-target population most strongly in terms of frequency of the gene-drive allele. Notably, if *c* = 1, then $q_{2}^{*}$ falls sharply for *s* > s* and less so for *s* < s* (Fig 3D–3F), suggesting that deviation from s* is not symmetric in its impact on deme 2.

Similar results in terms of existence of DTEs, m*, and $q_{2}^{*}$ were obtained for the alternative model with the order of selection and migration reversed (Appendix B in S1 Text and S2 Fig). The values for the alternative model in Eq S2 only slightly differed from those for the model in Eq 3. In the models where conversion occurs after selection (Appendix C in S1 Text), we observe that DTEs are not attainable for any gene drive with *h* = 0 and *h* = 0.5, and that m* and $q_{2}^{*}$ are identical to those in models with conversion before selection for *h* = 1.

#### Exceeding the critical migration rate m*

For a given (*s*, *c*, *h*) configuration, the limiting migration rate at which the possibility for differential targeting can withstand migration is m*. However, we must also consider the consequences of applying gene drives when the migration rate exceeds m*, because the migration rate may unexpectedly change over time and may be difficult to measure. For a given configuration, if *m* > m*, then the two-deme system is expected to rapidly converge either to global loss or to global fixation of the gene-drive allele. A gene-drive configuration that converges to global loss if m* is exceeded results in a failure of the gene-drive application. This case would, in general, be preferable to a gene drive that converges to global fixation when migration exceeds m*, as the latter case would likely result in severe consequences for the non-target population.

To explore the outcomes for exceeding the threshold m*, we assume that when *m* changes, the system converges rapidly to its equilibrium state, and we ignore the transient dynamics of changes between stable states. With this assumption, we can consider the continuous changes in the stable equilibrium point in frequency space due to continuous changes in the migration rate. We track the trajectory of the DTE by varying *m* from 0 to m* for each gene-drive configuration (*s*, *c*, *h*). To investigate the consequence of a breach of m*, we continue this trajectory above m* with an instantaneous transition to either global fixation or global loss. The state to which the trajectory transitions was evaluated by determining whether $\left({\hat{q}}_{1},{\hat{q}}_{2}\right)$, computed for the DTE with *m* = m* − *ϵ*, lies in the basin of attraction of global loss or of global fixation for the DTE computed for *m* = m* + *ϵ*. For example, in Fig 2, the yellow DTE for *m* = 0.022, just below m*, is positioned in the red global-fixation basin of attraction for the scenario *m* = 0.023, just above m*. We therefore conclude that, for the configuration in Fig 2, the gene drive would converge to global fixation if m* were exceeded.

**Fig 4:**
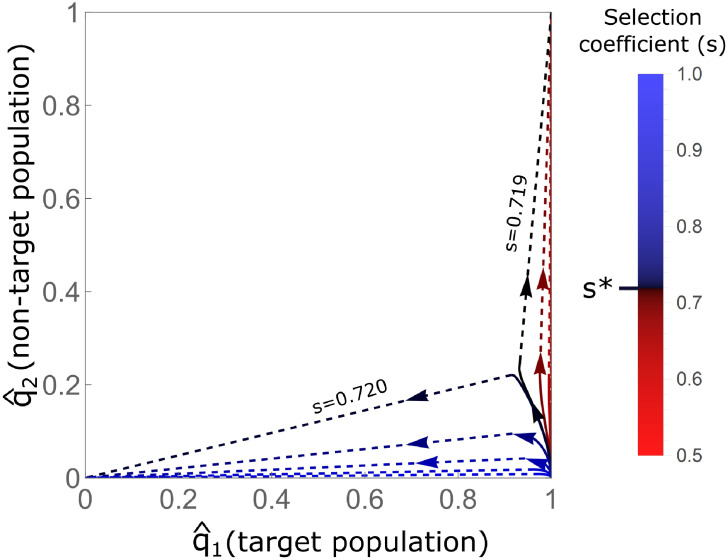
Trajectories of DTEs with increasing migration rates. Shown are trajectories for DTEs for gene drives with full conversion (*c* = 1; *h* values are not relevant in this case since there are no heterozygotes) and different selection coefficients (*s*), marked in different colors. Arrows show the direction of increased migration; solid lines show the increase of migration below m*, and dashed lines show the abrupt transition of the equilibrium state when m* is exceeded, to either global fixation (1, 1) or global loss (0, 0). The color bars for *s* are centered such that the selection coefficient that maximizes m*, s* ≈ 0.72, is shown in black, selection coefficients below s* are in red, and those above s* are in blue. s* forms the threshold between convergence to global fixation and convergence to global loss when the threshold m* is exceeded.

We computed trajectories of DTEs with an increase in migration rates for *c* = 1 and different selection coefficients *s* (Fig 4). We observe that for selection coefficients *s* lower than s*, exceeding m* results in global fixation, whereas for selection coefficients higher than s*, breaching the m* threshold results in global loss. This sharp transition, and the severity of the consequences of global fixation, suggest that a safer gene-drive configuration is one with a somewhat higher selection coefficient than s*; maintaining a safe distance from this sharp transition would be prudent, although at a cost of producing a decrease in m*. The qualitative behavior of the system with respect to breaching m* is similar for *c* < 1, with a different threshold selection coefficient *s* that distinguishes global fixation from global loss.

#### Perturbations from the DTEs

So far, we have evaluated gene-drive spillovers by considering the equilibria to which the dynamics converge deterministically. However, we have not considered the possibility that stochastic events and processes might destabilize the system, allowing it to escape the DTE due to perturbations, and to reach a different stable state. In this section, we consider two types of perturbations: (1) perturbations due to genetic drift, and (2) perturbations due to an external event, such as a significant ecological disturbance, which can affect the allele frequencies in the demes.

**Fig 5:**
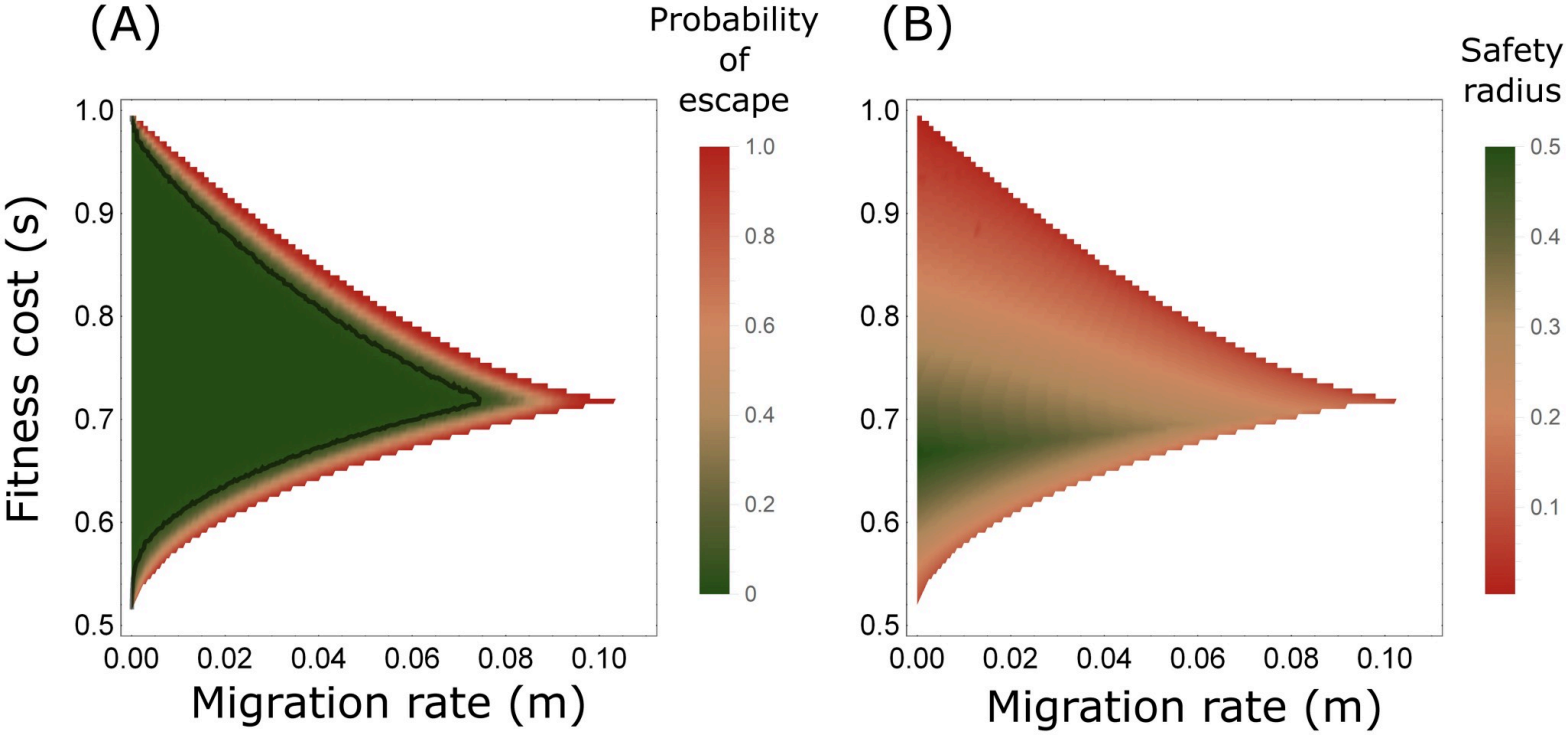
Perturbation from DTEs. Results shown for gene drives with full conversion, *c* = 1, and for different migration rates *m* and selection coefficients *s*. White regions denote scenarios for which *m* > m*. (A) The probability of escape from the DTE due to genetic drift, defined as the probability of departing from the attraction basin of the DTE over 100 generations with genetic drift, in a Wright-Fisher population with *N*_*e*_ = 100. The black line denotes 5% probability of escape. Probabilities were estimated with 1000 simulated replicates. (B) The safety radius of the DTE.

The impact of genetic drift on the probability of escaping a DTE depends on the effective population size of each deme, *N*_*e*_. We estimated the *probability of escape*—the probability of leaving the basin of attraction of the DTE in 100 generations, starting at the DTE—by simulating the dynamics of models similar to Eq 3, but with allele frequencies also affected by genetic drift (see Appendix D in S1 Text for details). We examined the scenario of *N*_*e*_ = 100 and *c* = 1 as an example (recall that *h* has no significance for *c* = 1 because there are no heterozygotes). In general, for this scenario, the probability of escape remains below 5% until m* is approached (Fig 5A and S10(A) Fig). For larger populations, with *N*_*e*_ > 100, the probabilities of escape are expected to be lower than those in Fig 5A and S10(A) Fig due to weaker genetic drift, whereas for populations with *N*_*e*_ < 100, the probabilities of escape are higher (S11 Fig).

Genetic drift is a source of perturbation that is intrinsic to the system, but we also consider extrinsic perturbations that may alter allele frequencies in the demes. In this case, we evaluate the resilience to perturbations by examining the effect of a single perturbation on the eventual DTE of the system. We define the safety radius of a gene-drive configuration under a given migration rate to be the maximal magnitude in the allele frequency space of a perturbation from a DTE above which the system will not converge back to the DTE, i.e., the shortest Euclidean distance from the DTE to the boundary of its basin of attraction (details in Appendix D in S1 Text). For perturbations from the DTE larger than the safety radius, the system is expected to converge to an equilibrium that is not a DTE. Similar to the probability of escape from the DTE by genetic drift, the safety radii are particularly small when migration rates approach m* (Fig 5B and S10(B) Fig).

#### Asymmetric migration

The analysis above has been conducted under the assumption that migration between the two populations is symmetric. If outgoing migration is assumed to be proportional to the population size or density, then the symmetry assumption could be interpreted as an implicit assumption of equal population sizes. In this section, we explore the effect of asymmetry in migration by supposing that the parameter *m* represents the migration rate from deme 2, the non-target population, to deme 1, the target population, and that migration from deme 1 to deme 2 occurs at rate *am*. Therefore, *a* is the ratio between the migration rates in the two directions. Analogously to Eq 2, the post-migration gene drive frequencies are given by:

$$\begin{array}{c} \begin{array}{c} {\tilde{q}}_{1}=[\left(1-am\right)q_{1}+mq_{2}]\frac{1}{1-am+m}\\ {\tilde{q}}_{2}=[\left(1-m\right)q_{2}+amq_{1}]\frac{1}{1-m+am}\;, \end{array} \end{array}$$ (4)

where in each equation, the frequencies are normalized to account for the differences in the two migration rates under the assumption that population sizes are density-regulated and are fixed. To avoid nonsensical migration rates, we only address scenarios for which *am* < 1. We obtain the equilibrium conditions for this asymmetric two-deme model by solving Eq 3, using Eq 4 instead of Eq 2 for the post-migration frequencies. With *a* = 1, the asymmetric model reduces to the symmetric model (Eq 2).

Analogously to the symmetric model, we define $m_{a}^{*}\left(s,c,h\right)$ as the critical migration rate for existence of DTEs in the model with asymmetric migration ratio *a*. Equivalently, we define $q_{2,a}^{*}\left(s,c,h\right)$ as the maximal DTE frequency in the non-target population with asymmetric migration ratio *a*. Note that $m_{a}^{*}$ describes the critical migration rate in terms of migration from deme 2 to 1; at the critical threshold, migration from deme 1 to 2 is, therefore, $am_{a}^{*}$.

**Fig 6:**
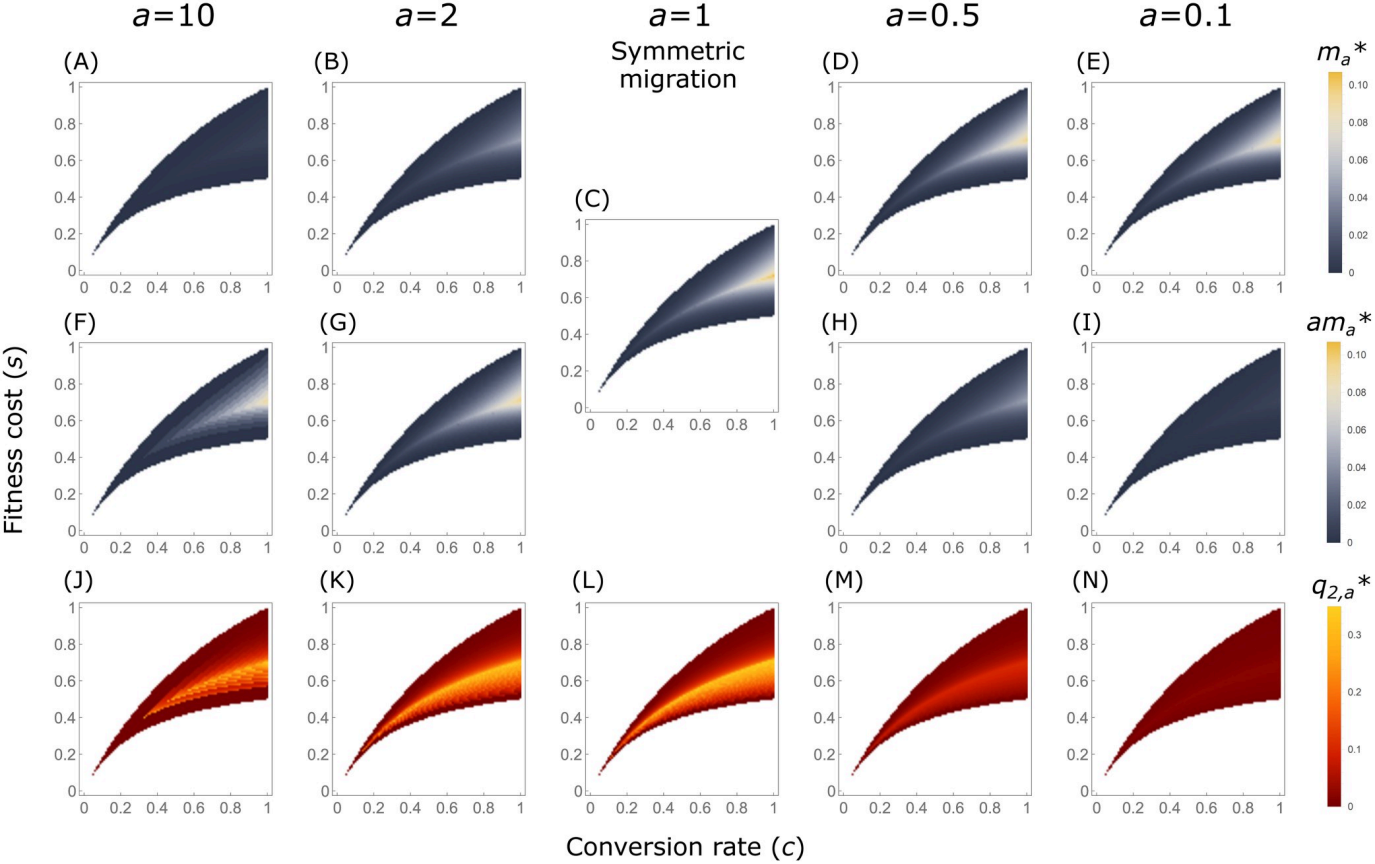
Critical migration thresholds for differential targeting, $m_{a}^{*}$, and maximal gene drive frequencies in the non-target population, $q_{2,a}^{*}$, with asymmetric migration, for additive gene drives (*h* = 0.5). (A–E) Critical migration thresholds from the non-target deme to the target deme, $m_{a}^{*}$, for migration ratio *a*. (F–I) Critical migration thresholds expressed as migration from target to non-target demes, $am_{a}^{*}$, for migration ratio *a*. (J–N) Maximal gene-drive frequencies in the non-target population at the at DTE, $q_{2,a}^{*}$, with migration ratio *a*.

We numerically computed $m_{a}^{*}$ and $q_{2,a}^{*}$ for different migration ratios, *a* = 10, 2, 0.5 and 0.1, and for different gene drive configurations (Fig 6 for additive gene drives with *h* = 0.5; results for *h* = 0 and *h* = 1 are presented in Appendix E in S1 Text and S12 and S13 Figs). For *a* = 10 and *a* = 2, we observe that $am_{a}^{*}<1$ for all gene drive configurations we examined, ensuring that the scenarios we analyze are valid.

When migration rates are lower from the non-target population to the target population (*a* > 1), the critical thresholds $m_{a}^{*}$ are substantially lower than in the symmetric case (Fig 6A–6B compared to Fig 6C). However, in terms of migration rates from the target to non-target population, the thresholds $am_{a}^{*}$ are fairly similar to the thresholds in the symmetric case (Fig 6F–6G compared to Fig 6C), although slightly lower; the maximal critical threshold across the parameter space for $10m_{10}^{*}$ is 0.090 and for $2m_{2}^{*}$ is 0.096, compared to 0.110 for the symmetric case $m_{1}^{*}$. The opposite pattern is observed with *a* < 1, where $m_{a}^{*}$ values are similar to, and slightly lower than, $m_{1}^{*}$ values across the gene drive configuration space (Fig 6D–6E compared to Fig 6C), but $am_{a}^{*}$ values are much lower (Fig 6H–6I compared to Fig 6C). These results indicate that for a given gene drive configuration (*s*, *c*, *h*), maintaining DTEs and avoiding spillovers in a two-deme system with asymmetric migration in which the higher of the two rates is *m*, is comparable to, but slightly more difficult than, in the symmetric case with migration rate *m*. This does not depend on which migration rate is larger.

The maximal impact of the DTE on the non-target population ($q_{2,a}^{*}$) follows the critical thresholds in terms of migration from the target to the non-target, $am_{a}^{*}$, with substantially lower impact for *a* < 1 and slightly lower impact for *a* > 1, compared to the symmetric case (Fig 6J–6N). Therefore, reduced migration from the target population to the non-target population (lower *a* values) would result in substantially reduced impact of the gene drive on the non-target population at the DTE.

### Gene drive design for applications in nature

Although gene-drive applications are not yet at the stage of deployment in natural settings, it is useful to understand spillover dynamics in specific applications that are already at the design and experimentation stages. Obtaining information on some of the parameters in our models, such as *s* or *m*, for specific systems in natural settings is challenging. However, using available information and making simplifying assumptions to demonstrate the application of the framework in different settings can give insight into the type of considerations that should be taken into account when designing system-specific gene drives. Here, we use two examples to illustrate how the models might behave in the context of scenarios of interest.

#### Malaria vectors

One of the most discussed potential applications of gene drives is for the control of malaria vectors—*Anopheles* mosquitoes [41–43]. Before the deployment of gene drives in malaria-affected regions, field trials in natural but controlled settings will likely occur [44, 45]. These field trials could, for example, be conducted on islands near the coast of sub-Saharan Africa, in order to simulate conditions similar to those found on the African mainland but in a contained setting [45].

To demonstrate how our theory can be applied, we consider such a field trial, on an island where *Anopheles* mosquitoes occur naturally. Biosafety measures for such an experiment should account for the possibility that mosquitoes carrying the gene-drive allele would find their way to the mainland [45], either through migration processes similar to those that resulted in the initial colonization of the island [46, 47], or through human-assisted migration (e.g., hitchhiking on human transportation).

For *Anopheles* mosquitoes, high gene-drive conversion rates have been reported, approaching full conversion, *c* = 1 [48]. We adopt the same conversion rate, *c* = 1, for our hypothetical field trial example. To achieve DTEs, we must ensure that the gene-drive configuration is within *B*_2_.

For *c* = 1, irrespective of *h*, the gene-drive configuration is in *B*_2_ when *s* > 0.5 (S3 Fig, red regions for *c* = 1). For *s* < 0.5, the gene-drive configuration is in *A*_1_ (S3 Fig, green regions for *c* = 1), and the gene drive is predicted to reach fixation in both populations at any migration rate, perhaps even if only a single individual migrates to the mainland. Therefore, to prevent spillovers and substantial impacts of the gene drive outside the target island, it is crucial to ensure that the fitness of homozygotes of the gene-drive allele, 1 − *s*, is at most half that of the wild types. This choice ensures that the gene-drive configuration is in *B*_2_.

Because it is likely that the migration rate is not accurately known, increasing the critical migration threshold increases the likelihood that DTEs can be maintained under unknown migration rates. For full conversion, *c* = 1, our results indicate that the maximal critical migration threshold attainable is m* ≈ 0.1, attained for some intermediate fitness cost s* (Fig 3A–3C, yellow bands for *c* = 1). With this configuration, it is possible for the gene drive to be maintained at low frequency on the mainland, even if connectivity between the island and the mainland is as high as *m* ≈ 0.1. For migration rates above this critical migration threshold, DTEs no longer exist. Given uncertainty in migration rates, it is therefore prudent to design the gene drive with a fitness cost close to s*. Our model can provide an initial estimate of s*, but this parameter would likely need to be ascertained from lab experiments to account for the many biological aspects we do not model here.

Our results for *c* = 1 indicate that when m* is exceeded, the gene drive is globally driven to fixation for *s* < s*, whereas for *s* > s* it is globally driven to loss (Fig 4). It is, therefore, advisable to ensure that *s* is slightly larger than s*, as an additional measure to prevent spillover to the mainland. Hence, if migration exceeds the migration limit (e.g. Appendix F in S1 Text and S14 Fig) because of underestimation of the actual migration rates or changes in those rates, the gene drive will fail to spread in both the island and the mainland, rather than spreading in both.

For the configuration *c* = 1 and *s* = s*, initiating the gene drive so it converges to the DTE requires a high initial gene-drive frequency on the island, particularly if connectivity to the mainland is substantial; for example, in S12 Fig, the intersection of the border between the yellow and blue regions with the *x*-axis is at *q*_1_ = 0.63 for *m* = 0, and this intersection point increases for higher migration rates. Attaining such high initial frequencies would require releasing many genetically modified individuals, but could perhaps be feasible for a field trial on an island.

#### Invasive rodents

As a second example, we consider the deployment of gene drives for control of invasive rodents on islands [15, 43]. Here, the consequences of spillovers are critical not only for field trials, but also for the intended purpose of the gene drive, because our final aim is to control the species only on specific islands, without influencing the species in its native range [20, 21, 49]. CRISPR-based gene drives have been shown to work in mice, with lower conversion effectiveness than that reported in invertebrates [40]. For this example, we assume that a gene drive has been developed for the relevant rodent species with *c* = 0.72, the highest conversion rate reported in mice to date [40].

In this case, unlike for the malaria-vector example with *c* = 1, we must also consider the dominance parameter *h*. For a recessive gene drive (*h* = 0), DTEs are possible for gene-drive configurations with 0.5 < *s* < 0.73 (S3 Fig, red region for *c* = 0.72 and *h* = 0). Within this range of *s*, the DTEs can be maintained under the highest migration rate of m* = 0.02 (Fig 3A, pale yellow band for *c* = 0.72 around *s* = 0.6). This critical migration threshold is substantially lower than could be attained in the mosquito example above, meaning that under ideal gene drive configurations, differential targeting can be maintained under higher connectivity in mosquitoes than in rodents. For an additive gene drive (*h* = 0.5), the fitness-cost range for DTEs is wider, 0.45 < *s* < 0.84 (S3 Fig, red region for *c* = 0.72 and *h* = 0.5), and DTEs can be maintained up to a higher critical threshold of m* = 0.06 (Fig 3B, pale yellow band for *c* = 0.72). For a dominant gene drive (*h* = 1), the fitness-cost range is even wider, 0.42 < *s* < 1 (S3 Fig, red region for *c* = 0.72 and *h* = 1), and maximal critical thresholds are even higher, m* = 0.08 (Fig 3C, pale yellow band for *c* = 0.72). Therefore, unlike in the mosquito example, for rodents we see that *h* plays an important role in determining the parameter ranges in which DTEs can be attained, as well as the critical migration thresholds for DTEs. In this particular example, the critical migration limit is more than three times higher for a dominant gene drive than for a recessive one. Notably, current mouse gene drives are more efficient when conversion occurs in the germline rather than in the zygote [40]. Our analysis of gene drives with germline conversion (Appendix C in S1 Text) indicates that *h* > 0.5 is required for DTEs to exist, reinforcing the conclusion that dominance of the gene drive allele will likely be crucial for differential targeting in rodents.

For any analysis of gene-drive spillovers, it is important to identify the relevant scale for which the migration rates *m*, defined as migration probabilities of individuals, apply. These rates refer to populations in the region from which migration occurs. In our example, considering a target island with an invasive rodent species and a non-target island with native rodents, and assuming that the rodents migrate mostly via a major entry point on these islands, such as a main port, the subpopulation of the port and its immediate surroundings, and not the population of the entire island, would likely be the appropriate unit for estimating *m*. Migration rates among subpopulations on an island typically exceed migration rates between populations on different islands. Therefore, for migration between two highly-populated islands, for example, if we measure *m* using the entire population of each island, and not among appropriate subpopulations, then we might significantly underestimate the appropriate *m* for the application of our models.

## Discussion

We have explored the possibility of effectively applying a gene drive in a target population while limiting the exposure of a non-target population to spillovers. By investigating equilibria of the evolutionary dynamics, we have shown that with some gene-drive configurations (*s*, *c*, *h*) for which a polymorphic unstable equilibrium exists in the one-deme model (*B*_2_ configurations), it is possible to initiate the gene drive such that differential targeting—higher equilibrium gene-drive allele frequencies in the target population than in the non-target population—is possible. However, we have also shown that for these configurations, upon increasing the migration rate *m*, a sharp transition occurs to a state in which the DTE no longer exists, and the two populations face similar fates, either global loss or global fixation of the gene-drive allele. We also showed that critical migration thresholds are highest for a full conversion gene drive (*c* = 1) with some intermediate fitness cost s*.

For most *B*_2_ configurations, other than in a narrow region of the parameter space for (*s*, *c*, *h*), differential targeting can be achieved only for low migration rates (blue regions in Fig 3A–3C). This observation was seen in two-deme models with different assumptions regarding migration asymmetry and timing of migration, selection, and conversion (Appendices B, C, and E in S1 Text). Consequently, for relatively high migration rates, it is unlikely that gene drives could be configured with enough accuracy to provide sufficient confidence that differential targeting could be achieved. In general, our analysis suggests that gene-drive spillovers to the non-target population should therefore be considered likely, and prevention of spillovers should in most cases not rely on differential targeting. Only when the gene-drive configuration (*s*, *c*, *h*) and ecological circumstances have been validated with high accuracy, or when low migration rates between populations can be assured, will it be possible to view differential targeting as a practical measure for limiting spillovers.

We found that the configuration that maximizes the critical migration threshold m* is also the one that maximizes the impact on the non-target population in the sense of producing the largest $q_{2}^{*}$ at equilibrium (Fig 3D–3F). Allowing the selection coefficient to vary, this same configuration also differentiates between scenarios for which increasing migration above m* results in global loss or global fixation of the gene-drive allele (Fig 4). In practical settings, such breaches of the critical migration threshold may occur as a result of unexpected increases in migration, or from errors in approximating actual migration rates. As a precaution, it would be wise to design gene drives (*s*, *c*, *h*) for which exceeding m* results in global loss rather than global fixation, in order to avoid full exposure of the non-target population to the gene drive.

Many mathematical gene-drive modeling efforts so far have followed classic population-genetic models in which allele frequencies do not directly effect the population size (but see [50]). While this assumption is appropriate for most loci of interest in population genetics, gene drives are designed specifically to violate this assumption on very short time-scales. For example, our models assume fixed migration rates, but if a DTE is attained and the gene drive reaches high frequencies in the target population, population-size decline in the target population would likely result in decreased migration from target to non-target populations. Although our models do not directly incorporate the effect of gene-drive frequencies on population dynamics, we can use our investigation of asymmetric migration to provide some insights. Under differential targeting, we expect reduced outgoing migration from the target population but not from the non-target population, because only the target population is substantially affected by the gene drive on a population level. In terms of our model, this reduced migration from the target to non-target populations would result in a decrease in the migration ratio *a* from its initial value. Therefore, with the reservation that we solve for equilibrium conditions and not for dynamic decrease in migration, the observation that for given gene-drive configurations, critical thresholds are not breached as *a* decreases (Fig 6A–6I) implies that differential targeting may persist through the formation of asymmetric migration. In addition, we observe that as *a* decreases, the maximal impact on the non-target population ($q_{2,a}^{*}$) decreases (Fig 6J–6N). Therefore, differential targeting may be a relevant strategy even when considering the demographic impact of gene drives on the target population.

For the two potential applications of gene-drive deployment, malaria-vector field trials and invasive rodents on islands, we observed that the conversion efficiency in the organism affects the design configurations for limiting spillovers. For very high conversion rates, as have been attained in mosquitoes, the main consideration is the selection coefficient of the gene drive, and migration limits for maintaining differential targeting can be relatively high. However, in the case of rodents, where conversion efficiency is currently lower, we see that the degree of dominance of the gene-drive allele has a strong effect on the migration limits. The maximal migration limits are lower than for the mosquito example, with higher dominance of the gene-drive allele resulting in higher migration limits. This result suggests that, with minimizing the risk of spillover as a goal, dominance should be given a priority in gene-drive design for rodents, while it is not as important for malaria vectors, particularly for gene-drive conversion in the germline. These examples also highlight that the gene-drive selection coefficient should be chosen with the other parameters, particularly conversion rates, in mind.

Before CRISPR-based gene drives can be efficiently and safely applied, many challenges, such as the potential evolution of resistance to the gene-drive modification [51–57], must be overcome. For the type of differential targeting described here, an accurate configuration of gene-drive parameters, *s*, *c*, and *h*, is required. However, it is unclear whether such accuracy is feasible [10, 40, 48, 58–61]. Another difficulty for differential targeting is that implementation might require a concentrated effort to initiate gene drive deployment in the basin of attraction of the desired equilibrium (the DTE). For example, in the malaria-vector field trial case examined here, the gene-drive allele frequency must be pushed above 0.6 before the system is expected to converge to the DTE. This effort would therefore involve engineering and releasing a large and potentially unfeasible number of engineered individuals. A third problem is that we do not yet understand the consequences of more complex population structure, involving more than two populations or populations that are internally structured. Given these difficulties, to understand and mitigate the dangers of spillovers, it will be important to study more elaborate population structures in models of gene drives.

Several approaches have been recently proposed for mitigating spillovers, involving complex gene drive architectures and deployment strategies [39, 62–69], as well as for implementing countermeasures to halt an ongoing gene drive [10, 70–72]. A few of these gene-drive architectures have been demonstrated in laboratory settings [10, 38, 53, 60, 73]. However, performance of mitigation strategies in local confinement of a gene drive has only been examined through proof-of-concept mathematical models, and their behaviors outside the specific population and environmental conditions examined are still not well understood. We have investigated the prospects of differential targeting as a confinement strategy under a standard homing CRISPR-based gene drive construct, but our model could potentially be extended to incorporate more complex constructs, and be deployed under various strategies. Therefore, given the dangers and many unknowns of gene-drive dynamics, we suggest that multiple safeguards should be used in parallel. In this way, differential targeting, sophisticated genetic architectures, informed deployment strategies, and countermeasures for halting the spread of already-active gene drives could be used in concert to reduce gene-drive spillover risks.

Mathematical investigations of the type conducted here are intended to reveal qualitative behaviors of expected dynamics, and can provide directions for mitigating gene drive spillovers by configuring gene-drive parameters. However, the many simplifying assumptions of these models mean that any conclusions, particularly those involving specific parameter values, need to be thoroughly explored in more realistic models, in lab experiments, and ideally in natural conditions, before being considered viable options for gene-drive deployment. In addition, we have studied equilibrium conditions to understand the direction to which two-deme systems would tend, but we did not study explicit transient dynamics, which are important for understanding, for example, how long it would take for the system to converge to equilibria. Therefore, to improve our understanding of gene drive spillovers, further exploration of gene-drive models incorporating various gene-drive constructs, deployment strategies, and ecological features, as well as lab experimentation with structured caged populations, is needed.

The existence of scenarios permitting differential targeting should not be read as a call for applications of gene drives in wild settings, nor as a blueprint for CRISPR-based gene-drive design. Instead, we believe the consequences of gene-drive application and the potential for spillovers should be further discussed in the scientific community and in society at large before actual gene-drive applications are realized, and a goal of this paper is to facilitate and inform these discussions.

## Methods

### Equilibria

Equilibria were computed by numerically solving the systems of equations in Eq 3, with the equilibrium conditions $q_{1}^{\prime }=q_{1}$ and $q_{2}^{\prime }=q_{2}$, using Mathematica software [74].

### Stability of equilibria

For each equilibrium $\left({\hat{q}}_{1},{\hat{q}}_{2}\right)$, its stability was determined by examining how the trajectories of the allele frequencies in the two populations behave in its vicinity in allele frequency space.

The system of discrete-time recursion equations that describe the change of allele frequencies over a single generation are:

$$\begin{array}{c} \begin{array}{c} q_{1}^{\prime }=f_{1}\left(q_{1},q_{2}\right)\\ q_{2}^{\prime }=f_{2}\left(q_{1},q_{2}\right), \end{array} \end{array}$$ (5)

where *f*_*i*_ (for *i* = 1, 2) is defined in Eq 3 or Equation S2 in S1 Text. To analyze the stability of the system in the vicinity of an equilibrium $\left({\hat{q}}_{1},{\hat{q}}_{2}\right)$, we linearized the nonlinear functions *f*_1_ and *f*_2_ by taking the first-order approximation of the Taylor expansion about the equilibrium. From this linearization, in order to understand the consequences of small perturbations from the equilibrium, we examined the matrix of the partial derivatives of *f*_1_ and *f*_2_, evaluated at the equilibrium (i.e., the Jacobian matrix of Eq 5):

$$\begin{array}{c} J=[\begin{array}{cc} \frac{\partial f_{1}}{\partial q_{1}} & \frac{\partial f_{1}}{\partial q_{2}}\\ \frac{\partial f_{2}}{\partial q_{1}} & \frac{\partial f_{2}}{\partial q_{2}} \end{array}]. \end{array}$$ (6)

The local stability of the equilibrium point $\left({\hat{q}}_{1},{\hat{q}}_{2}\right)$ is determined by the two eigenvalues λ_1_ and λ_2_ of **J** evaluated at $\left({\hat{q}}_{1},{\hat{q}}_{2}\right)$. If both eigenvalues have modulus less than 1 (|λ_1_| < 1 and |λ_2_| < 1), then the equilibrium point $\left({\hat{q}}_{1},{\hat{q}}_{2}\right)$ is stable. If at least one eigenvalue has modulus greater than 1, then the equilibrium point is unstable [75]. For the special case with |λ_1_| = 1 or |λ_2_| = 1, see [75].

To generate Fig 2 and S4–S9 Figs, we computed the equilibria and determined their stability for all (*s*, *c*, *h*) combinations, with *s* and *c* ranging from 0 to 1 in increments of 0.01, and *h* = 0, 0.5 or 1. To generate Figs 3 and 4, these configurations were examined for each migration rate *m* between 0 and 0.15 at increments of 0.0001. Basins of attraction in Fig 2 and S14 Fig were computed by iterating the recursive equations with different initial conditions to identify the equilibrium to which they converge.

For a stable equilibrium, there exists a basin of attraction in allele frequency space around the equilibrium [76], such that any trajectory starting within the basin eventually converges to the stable equilibrium point. To determine basins of attraction for a configuration (*s*, *c*, *h*) and migration rate *m*, we started from a grid of points in frequency space, and for each point (*q*_1_, *q*_2_), we tracked its trajectory for 1000 generations by iterating Eq 3. We denote the endpoint of the trajectory with initial condition (*q*_1_, *q*_2_) by (*q*_1_, *q*_2_)_1000_. The attractor associated with (*q*_1_, *q*_2_) was defined as the equilibrium $\left({\hat{q}}_{1},{\hat{q}}_{2}\right)$ that was at a Euclidean distance less than 0.001 in frequency space from (*q*_1_, *q*_2_)_1000_. If there were no such equilibria or more than one such equilibrium, then the attractor of (*q*_1_, *q*_2_) was left undefined. The basins of attraction were computed for a grid of (*q*_1_, *q*_2_) points at a resolution of 0.01 × 0.01 in frequency space. For all computations used to generate Figs 2, 4, and 5, attractors were defined for all points computed.

## Supporting information

S1 Text Section A explains the evolutionary dynamics of a gene drive in a one-deme model. Section B contains a mathematical model and numerical results in the two-deme model with selection before migration. Section C contains a mathematical model and results in the two-deme model with conversion after selection and migration. Section D details the mathematical modeling and simulation procedures for adding genetic drift and computing the safety radius. Section E presents the results for recessive and dominant gene drives with asymmetric migration. Section F presents an analysis of the malaria-vector example.
(PDF)

S1 Fig Schematic depiction of a two-deme gene-drive model with selection occurring before migration. For interpretation of arrow colors, see Fig 1 in the main text.
(PNG)

S2 FigCritical migration thresholds for differential targeting in the two-deme model with selection before migration.(A–C) Maximal migration rates m* for which a differential-targeting equilibrium (DTE) exists. The colored regions denote the configurations for which a DTE exists (*B*_2_ configurations), and the regions in white denote configurations for which differential targeting of the demes is not possible (*A*_1_, *A*_2_, and *B*_1_). Over most of the parameter space, a DTE exists only for low migration rates (in blue), and only in a narrow band do DTEs exist with migration rates above m* = 0.05 (light yellow). (D–F) Maximal gene-drive frequencies in the non-target population, $q_{2}^{*}$, at differential-targeting equilibria (DTEs). $q_{2}^{*}$ values are correlated with m* values. The figure design follows Fig 3.(PNG)

S3 FigSubsets of gene-drive configurations, according to results derived from the one-deme model (Eq 1).Green: *A*_1_ configurations, with two trivial equilibria, $\hat{q}=0$ (loss) unstable and $\hat{q}=1$ (fixation) stable; yellow: *A*_2_ configurations, with two trivial equilibria, $\hat{q}=0$ stable and $\hat{q}=1$ unstable; blue: *B*_1_ configurations, with 1 stable non-trivial equilibrium and 2 unstable trivial equilibria; red: *B*_2_ configurations, with 1 unstable non-trivial equilibrium and 2 stable trivial equilibria. The panels show results for 11 values of *h* and all possible values of *s* and *c*.(PNG)

S4 FigPartitioning of the parameter space by the characterization of the equilibria for the two-deme migration-before-selection model (Eq 3), for recessive gene drives (*h* = 0), under different migration rates.Green—9 equilibria, 4 stable and 5 unstable, one of which is a DTE (stable and ${\hat{q}}_{1}>{\hat{q}}_{2}$); purple—9 equilibria, 8 unstable and 1 stable and symmetric (${\hat{q}}_{1}={\hat{q}}_{2}$); blue—5 equilibria, 2 stable trivial (global fixation and global loss) and 3 unstable; red—3 equilibria, 2 stable trivial and 1 unstable; orange—3 equilibria, 2 unstable trivial and 1 stable symmetric; white—2 trivial equilibria, 1 stable and 1 unstable (*A*_1_ and *A*_2_ configurations). In the bottom white region, the stable equilibrium is global fixation of the gene-drive allele (*A*_1_ configuration), and in the top white region, the stable equilibrium is global loss (*A*_2_ configuration). The only gene-drive configurations for which differential targeting is possible appear in green.(PNG)

S5 FigPartitioning of the parameter space by the characterization of the equilibria for the two-deme migration-before-selection model (Eq 3), for additive gene drives (*h* = 0.5), under different migration rates.Green—9 equilibria, 4 stable and 5 unstable, one of which is a DTE (stable and ${\hat{q}}_{1}>{\hat{q}}_{2}$); purple—9 equilibria, 8 unstable and 1 stable and symmetric (${\hat{q}}_{1}={\hat{q}}_{2}$); blue—5 equilibria, 2 stable trivial (global fixation and global loss) and 3 unstable; red—3 equilibria, 2 stable trivial and 1 unstable; white—2 trivial equilibria, 1 stable and 1 unstable (*A*_1_ and *A*_2_ configurations). In the bottom white region, the stable equilibrium is global fixation of the gene-drive allele (*A*_1_ configuration), and in the top white region, the stable equilibrium is global loss (*A*_2_ configuration). The only gene-drive configurations for which differential targeting is possible appear in green.(PNG)

S6 FigPartitioning of the parameter space by the characterization of the equilibria for the two-deme migration-before-selection model (Eq 3), for dominant gene drives (*h* = 1), under different migration rates.Green—9 equilibria, 4 stable and 5 unstable, one of which is a DTE (stable and ${\hat{q}}_{1}>{\hat{q}}_{2}$); purple—9 equilibria, 8 unstable and 1 stable and symmetric (${\hat{q}}_{1}={\hat{q}}_{2}$); blue—5 equilibria, 2 stable trivial (global fixation and global loss) and 3 unstable; red—3 equilibria, 2 stable trivial and 1 unstable; white—*A*_1_ configuration, with 1 stable (global fixation) and 1 unstable (global loss) equilibrium. The only gene-drive configurations for which differential targeting is possible appear in green.(PNG)

S7 FigPartitioning of the parameter space by the characterization of the equilibria for the two-deme selection-before-migration model (Eq S2), for recessive gene drives (*h* = 0), under different migration rates.Green—9 equilibria, 4 stable and 5 unstable, one of which is a DTE (stable and ${\hat{q}}_{1}>{\hat{q}}_{2}$); purple—9 equilibria, 8 unstable and 1 stable and symmetric (${\hat{q}}_{1}={\hat{q}}_{2}$); blue—5 equilibria, 2 stable trivial (global fixation and global loss) and 3 unstable; red—3 equilibria, 2 stable trivial and 1 unstable; orange—3 equilibria, 2 unstable trivial and 1 stable symmetric; white—2 trivial equilibria, 1 stable and 1 unstable (*A*_1_ and *A*_2_ configurations). In the bottom white region, the stable equilibrium is global fixation of the gene-drive allele (*A*_1_ configuration), and in the top white region, the stable equilibrium is global loss (*A*_2_ configuration). The only gene-drive configurations for which differential targeting is possible appear in green.(PNG)

S8 FigPartitioning of the parameter space by the characterization of the equilibria for the two-deme selection-before-migration model (Eq S2), for additive gene drives (*h* = 0.5), under different migration rates.Green—9 equilibria, 4 stable and 5 unstable, one of which is a DTE (stable and ${\hat{q}}_{1}>{\hat{q}}_{2}$); purple—9 equilibria, 8 unstable and 1 stable and symmetric (${\hat{q}}_{1}={\hat{q}}_{2}$); blue—5 equilibria, 2 stable trivial (global fixation and global loss) and 3 unstable; red—3 equilibria, 2 stable trivial and 1 unstable; white—2 trivial equilibria, 1 stable and 1 unstable (*A*_1_ and *A*_2_ configurations). In the bottom white region, the stable equilibrium is global fixation of the gene-drive allele (*A*_1_ configuration), and in the top white region, the stable equilibrium is global loss (*A*_2_ configuration). The only gene-drive configurations for which differential targeting is possible appear in green.(PNG)

S9 FigPartitioning of the parameter space by the characterization of the equilibria for the two-deme selection-before-migration model (Eq S2), for dominant gene drives (*h* = 1), under different migration rates.Green—9 equilibria, 4 stable and 5 unstable, one of which is a DTE (stable and ${\hat{q}}_{1}>{\hat{q}}_{2}$); purple—9 equilibria, 8 unstable and 1 stable and symmetric (${\hat{q}}_{1}={\hat{q}}_{2}$); blue—5 equilibria, 2 stable trivial (global fixation and global loss) and 3 unstable; red—3 equilibria, 2 stable trivial and 1 unstable; white—*A*_1_ configuration, with 1 stable (global fixation) and 1 unstable (global loss) equilibrium. The only gene-drive configurations for which differential targeting is possible appear in green.(PNG)

S10 Fig Perturbation from DTEs for the model with selection before migration (Eq S3). Results shown for gene drives with full conversion, *c* = 1, and for different migration rates *m* and selection coefficients *s*. White regions denote scenarios for which *m* > m*. (A) The probability of escape from the DTE due to genetic drift, defined as the probability of departing from the attraction basin of the DTE over 100 generations with genetic drift, in a Wright-Fisher population with *N*_*e*_ = 100. The black line denotes 5% probability of escape. Probabilities were estimated from 1000 simulated replicates. (B) The safety radius of the DTE for the *m*-before-*s* model.
(PNG)

S11 Fig Impact of genetic drift on the probability of escape. The critical migration threshold m* for the two-deme model in Eq 3 with *c* = 1 is shown in red. Other curves show the threshold for probability of escape >0.05% for different effective population sizes of the demes, for the same model, computed with 1000 simulated iterations of Eq S3 for each gene-drive configuration (equivalent to black line in Fig 5A and S10A Fig). *N*_*e*_ = 200 in blue; *N*_*e*_ = 50 in yellow; *N*_*e*_ = 10 in purple. Critical migration thresholds are effectively lower with lower effective population sizes.
(PNG)

S12 FigCritical migration thresholds $m_{a}^{*}$ and impact on non-target population $q_{2,a}^{*}$ with asymmetric migration for recessive gene drives (*h* = 0).(A–E) Critical migration thresholds from the non-target deme to the target deme, $m_{a}^{*}$, for migration ratio *a*. (F–I) Critical migration thresholds expressed as migration from target to non-target demes, $am_{a}^{*}$, for migration ratio *a*. (J–N) Maximal gene-drive frequencies in the non-target population at the DTE, $q_{2,a}^{*}$, with migration ratio *a*.(PNG)

S13 FigCritical migration thresholds $m_{a}^{*}$ and impact on non-target population $q_{2,a}^{*}$ with asymmetric migration for dominant gene drives (*h* = 1).(A–E) Critical migration thresholds from the non-target deme to the target deme, $m_{a}^{*}$, for migration ratio *a*. (F–I) Critical migration thresholds expressed as migration from target to non-target demes, $am_{a}^{*}$, for migration ratio *a*. (J–N) Maximal gene-drive frequencies in the non-target population at the at DTE, $q_{2,a}^{*}$, with migration ratio *a*.(PNG)

S14 Fig Equilibria and basins of attractions for the malaria-vector example. Shown are results for the *m*-before-*s* model with a gene-drive configuration (*s*, *c*, *h*) of *c* = 1, *s* = 0.73 and arbitrary *h*, as per the malaria-vector example in the main text. The circles show the equilibria. The colored regions show the attraction basins, with the basin colors corresponding to the stable equilibria. The arrows show the vector field that describes the magnitude and direction of the change in allele frequencies at each point in frequency space. The differential-targeting equilibrium (DTE) is the stable yellow equilibrium point, which exists for migration rates lower than m* ≈ 0.093.
(PNG)
