# Supplementary material for: Designing gene drives to limit spillover to non-target populations
Source: PLoS Genet. 2021 Feb 25;17(2):e1009278. doi: 10.1371/journal.pgen.1009278 (PMC7943199; doi:10.1371/journal.pgen.1009278)
Supplement: S1 Text — Section A explains the evolutionary dynamics of a gene drive in a one-deme model. Section B contains a mathematical model and numerical results in the two-deme model with selection before migration. Section C contains a mathematical model and results in the two-deme model with conversion after selection and migration. Section D details the mathematical modeling and simulation procedures for adding genetic drift and computing the safety radius. Section E presents the results for recessive and dominant gene drives with asymmetric migration. Section F presents an analysis of the malaria-vector example. (PDF) [file pgen.1009278.s001.pdf]

Supplementary Material for Greenbaum *et al.*

Contents

Appendix A - One-deme model **1**

Appendix B - Two-deme model with selection before migration **2**

Model 2

Results 2

Appendix C - Two-deme model with conversion after selection **3**

Model 3

Results 3

Appendix D - Modeling genetic drift and safety radius **4**

Genetic drift 4

Safety radius 5

Appendix E - Asymmetric migration **6**

Appendix F - Malaria-vectors example **7**

Appendix A - One-deme model

In this Appendix we recall the formulation of the evolutionary dynamics of a gene drive in a one-deme model by a recursion equation [1, 2]. Assuming Hardy-Weinberg equilibrium, and denoting the gene-drive allele frequency by q, the frequencies of the genotypes AA, Aa, and aa are q^2, $2q(1-q)$, and $(1-q)^2$, respectively. We denote the allele frequency in the subsequent generation by q'.

The AA homozygote contributes fully to q', and is subject to a selection coefficient of $1-s$; in total, the relative contribution of AA genotypes to q' is $q^2(1-s)$ (red arrow in Fig 1A). Unconverted heterozygotes, Aa, contribute only half of the contribution of AA, since they pass on the A allele to offspring with probability 0.5. The unconverted heterozygotes represent a fraction $1-c$ of the total heterozygotes, and they are subject to selection coefficient $1-hs$; we let $s_n = 0.5(1-c)(1-hs)$, and the total relative contribution of unconverted heterozygotes is $2q(1-q)s_n$ (green arrow in Fig 1A). Converted heterozygotes represent a proportion c of the heterozygotes, but because they are converted to AA genotypes, they contribute fully to q', and the relative contribution of converted heterozygotes is $2q(1-q)s_c$. If conversion occurs before selection (in the zygote), then individuals experience selection as homozygotes with selection coefficient $1-s$. In this case, we let $s_c = c(1-s)$. If, on the other hand, conversion occurs after selection (in the germline), then individuals experience selection as heterozygotes with selection coefficient $1-hs$. In this case, we let $s_c = c(1-hs)$.

Combining the three cases and normalizing by the mean fitness in the population so that frequencies in the next generation sum to 1, we obtain the following recursion for the gene-drive allele frequency [1, 2] (identical to Eq 1):

$$q' = \frac{q^2(1-s) + 2q(1-q)(s_n + s_c)}{\overline{w}}, \tag{S1}$$

where the mean fitness is $\overline{w} = q^2(1-s) + 2q(1-q)(2s_n + s_c) + (1-q)^2$. The numerator in Eq S1 describes the genotype frequencies weighted by their relative fitnesses and their contributions to the A allele frequency (gray box in Fig 1A), and the denominator normalizes this relative contribution by the mean fitness in the population (orange arrow in Fig 1A).

Appendix B - Two-deme model with selection before migration

Model

In Eq 3, migration occurs before migration, and therefore selection acts on the offspring in the deme to which they migrated. Here, we develop an alternative model in which selection occurs before migration, meaning that density regulation acts on offspring in their parental deme. In this model, the population is sampled after migration occurs, whereas in the model in the main text, the population is sampled after selection occurs.

We determine the pre-migration frequencies of A by considering the relative contributions of the genotypes and normalizing by the mean fitness for each of the demes, as in Eq 1 (S1 Fig, orange arrows). These mean fitnesses, $\overline{w}_1$ and $\overline{w}_2$, are $\overline{w}_i = q_i^2(1-s)+2q_i(1-q_i)(2s_n+s_c)+(1-q_i)^2$. Next, we consider the impact of migration on the pre-migration gene pools of the demes, considering the relative proportion of migrants and residents, as in Eq 2 (S1 Fig, black arrows). Combining these two steps, we obtain:

$$
\begin{aligned}
q_1' &= (1-m)\frac{q_1^2(1-s)+2q_1(1-q_1)(s_n+s_c)}{\overline{w}_1} + m\frac{q_2^2(1-s)+2q_2(1-q_2)(s_n+s_c)}{\overline{w}_2} \\
q_2' &= (1-m)\frac{q_2^2(1-s)+2q_2(1-q_2)(s_n+s_c)}{\overline{w}_2} + m\frac{q_1^2(1-s)+2q_1(1-q_1)(s_n+s_c)}{\overline{w}_1}.
\end{aligned}
\tag{S2}
$$

The terms on the left in the sums represent the contributions of residents to the frequencies, and the terms on the right represent the contributions of migrants.

Results

Although minor differences exist between the outcomes for the two models we examined (the one in the main text and the one presented here), in general, the order of migration and selection has little effect on the properties we studied.

The migration thresholds m^* were almost identical in the two models. For Eq S2, m^* increases for higher conversion rates c, and it is maximal for $c = 1$ and $s^* \approx 0.72$, the same as for the model in Eq 3 (S2A–C Fig, Fig 3A–C). The maximal value is obtained at $m^* \approx 0.109$ in the model in Eq S2, compared to $m^* \approx 0.110$ in the model in Eq 3 (S2A–C Fig, Fig 3A–C).

The maximal allele frequencies in the non-target deme q_2^* show qualitatively similar patterns for the two models we examined, although q_2^* values were slightly higher in the model with selection acting before migration than in the model in Eq 3 (S2D–F Fig, Fig 3D–F). For example, for $c = 1$, we have $q_2^* = 0.34$ for the model in Eq S2, compared to $q_2^* = 0.28$ for the model in Eq 3 (S2D–F Fig, Fig 3D–F).

Appendix C - Two-deme model with conversion after selection

Model

The timing of the homing reaction with respect to gene expression is an important factor in gene drive design [1]. In the main text, we explored a model where migration occurs after conversion of gene drive heterozygotes aA to gene drive homozygotes AA, appropriate for genetic architectures in which conversion occurs in the zygote. However, some CRISPR-based gene drive designs involve conversion in the germline—i.e., the homing reaction is active after gene expression [1, 3, 4]. Therefore, in this section, we consider conversion occurring after migration and selection.

For such gene drives, the contribution of genotypes to the post-migration gene pool (or the pre-migration gene pool in Appendix B in S1 Text) is the same as in the model in Eq 1, except that converted and non-converted heterozygotes both experience selection as heterozygotes, $1 - hs$, as opposed to converted heterozygotes experiencing selection as homozygotes, $1 - s$. Therefore, to model conversion occurring after migration and selection, we can use the models developed in the main text and in Appendix B in S1 Text, and re-define $s_c = c(1 - hs)$, instead of $s_c = c(1 - s)$ [1].

Results

First, we note that for dominant gene drives ($h = 1$), heterozygotes experience the same selection as gene drive homozygotes. Therefore, whether conversion occurs prior to selection and migration or before selection and migration has no impact on the results. This is reflected in the fact that for $h = 1$, Eq 3 is the same for $s_c = c(1 - hs)$ and $s_c = c(1 - s)$. Therefore, because the two models converge for $h = 1$, the results in Fig 3C and F, S2C Fig, and S2F Fig also apply to the model with conversion occurring after selection and migration.

Second, we note that for recessive gene drives ($h = 0$), in the one-deme model, the only equilibrium solutions are in the sets A_1 (two equilibria with fixation stable) and B_1 (three equilibria with the non-trivial equilibrium stable), see Fig 1 ($h = 0$ panel) in [1]. For $h = 0.5$, the equilibria in the one deme model are in either A_1 or A_2 (two equilibria with loss stable), see Fig 1 ($h = 0.5$ panel) in [1]. In other words, recessive and additive gene drives in which conversion occurs after selection are not threshold-dependent, for any s and c values [1]. Because for $h = 0$ and $h = 0.5$, there are no configurations in B_2, which is required for existence of DTEs, differential targeting is not possible for any recessive or additive gene drive for which conversion occurs after selection and migration. In summary, the case of gene drive conversion in the germline is less amenable to differential targeting, except if the gene drive allele is dominant (at least to a degree) over the wild-type allele, in which case critical thresholds are similar to gene drives in which conversion occurs in the zygote.

Appendix D - Modeling genetic drift and safety radius

Genetic drift

To determine the probability of escape from DTEs, we formulated the model as a stochastic process. We used the deterministic Eq 3 to generate binomial distributions with $2N_e$ trials, following the Wright-Fisher model for diploid populations, for each population, with the same means as for Eqs 3 and S2 [5]. Genetic drift was implemented after both selection and migration, in both models.

The distribution of the gene-drive allele frequency following one generation in the two-deme model with migration occurring before selection (the model presented in the main text) is given by:

$$\begin{aligned} q_1' &\sim Binomial\left(2N_{e,1}\ ,\ \frac{\widetilde{q}_1^2(1-s)+\widetilde{q}_1(1-\widetilde{q}_1)(s_n+s_c)}{\overline{w}_1}\right)\times\frac{1}{2N_{e,1}} \\ q_2' &\sim Binomial\left(2N_{e,2}\ ,\ \frac{\widetilde{q}_2^2(1-s)+\widetilde{q}_2(1-\widetilde{q}_2)(s_n+s_c)}{\overline{w}_2}\right)\times\frac{1}{2N_{e,2}}, \end{aligned} \tag{S3}$$

where $N_{e,1}$ and $N_{e,2}$ are the effective population sizes in demes 1 and 2, respectively.

For the model with selection before migration presented in Appendix A in S1 Text, the distribution over one generation is given by:

$$\begin{aligned} q_1' &\sim Binomial\left(2N_{e,1}\ ,\ (1-m)\frac{q_1^2(1-s)+q_1(1-q_1)(s_n+s_c)}{\overline{w}_1}+m\frac{q_2^2(1-s)+q_2(1-q_2)(s_n+s_c)}{\overline{w}_2}\right)\times\frac{1}{2N_{e,1}} \\ q_2' &\sim Binomial\left(2N_{e,1}\ ,\ (1-m)\frac{q_2^2(1-s)+q_2(1-q_2)(s_n+s_c)}{\overline{w}_2}+m\frac{q_1^2(1-s)+q_1(1-q_1)(s_n+s_c)}{\overline{w}_1}\right)\times\frac{1}{2N_{e,2}}. \end{aligned} \tag{S4}$$

With these models, we can simulate the allele frequency trajectories in the two demes, accounting for genetic drift (results are shown in Fig 5A and S10A Fig). We generated trajectories for $N_{e,1} = N_{e,2} = 100$ starting at the DTE allele frequencies. We therefore consider the probability of escape once the DTE is reached, and not the probability of escape during the transient convergence to the stable state when the gene drive is initiated at allele frequencies other than the DTE frequencies. Results for the migration threshold for a probability of escape $> 0.05\%$, a stochastic equivalent of m^*, with different N_e values is shown in S11 Fig.

For each gene-drive configuration (s, c, h) and migration rate m, the equilibria and basins of attraction were determined. Next, for each (s, c, h, m), 1000 simulations of the stochastic process were initiated, starting at the DTE $(\hat{q}_1, \hat{q}_2)$, and they were iterated for 100 generations by repeatedly applying Eq S3 or S4. In each simulation, we determined whether or not the allele frequencies remained in the basin of attraction of the DTE during all 100 generations. The probability of escape was computed as the fraction of the simulations for which the allele frequencies did not remain in the attraction basin of the DTE. Trajectories that escaped the basin

of attraction at any time were considered as escaped trajectories, irrespective of whether or not they returned to the basin of attraction; this provides a conservative estimate of probability of escape.

Safety radius

In order to compute the safety radius for a given gene-drive configuration and migration rate, the equilibria and basins of attraction were first computed. The Euclidean distances, in frequency space, between the DTE and all points in its basin of attraction were then computed. The safety radius was defined as the infimum over distances, in frequency space, between the DTE and points outside its basin of attraction. In other words, for a DTE $(\hat{q}_1, \hat{q}_2)$ with basin of attraction $B_{(\hat{q}_1,\hat{q}_2)}$, the safety radius was defined as $\inf_{(q_1,q_2)\notin B_{(\hat{q}_1,\hat{q}_2)}} \sqrt{(\hat{q}_1 - q_1)^2 + (\hat{q}_2 - q_2)^2}$. In practice, the safety radius was determined by computing the minimal distance between the DTE and points outside the basin of attraction, for points at 0.01×0.01 resolution in frequency space. Results for the model with selection before migration appear in S10B Fig.

Appendix E - Asymmetric migration

In the main text, asymmetric migration for additive gene drives ($h = 0.5$) was analyzed (Fig 6). Here, we present the results for recessive (S12 Fig) and dominant (S13 Fig) gene drives with asymmetric migration. The results are qualitatively similar to those in discussed in the main text in terms of the effect of asymmetry on the critical migration thresholds (m_a^* and am_a^*) and the impact on the non-target population ($q_{2,a}^*$). As with additive gene drives, we observe that for $a > 1$, the critical thresholds in terms of migration from deme 2 (non-target) to 1 (target) are significantly lower than the symmetric migration case ($a = 1$), but are only slightly lower than the symmetric case in terms of migration from deme 1 to 2 (S12A–C, F–G Fig and S13A–C, F–G Fig). The opposite is observed for $a < 1$, where thresholds are substantially lower than for $a = 1$ in terms of migration from deme 1 to deme 2, but are only slightly lower in terms of migration from deme 2 to deme 1 (S12C–E, H–I Fig and S13C–E, H–I Fig). For both recessive and dominant gene drives, the impact of the DTE on the non-target population, $q_{2,a}^*$, follows the critical thresholds in terms of migration from the target to the non-target population, am_a^* (S12J–N Fig and S13J–N Fig).

Appendix F - Malaria-vectors example

Here we present an analysis to the one shown in Fig 2, except with the parameters of the Malaria-vectors example given in the main text, $c = 1$, and $s = 0.73$ (S14 Fig). The critical migration threshold for the malaria-vectors example is $m^* = 0.093$, which is higher then the threshold of $m^* = 0.022$ in the example in Fig 2.

To initiate a gene drive that will converge to the DTE, we need to drive the gene-drive allele frequencies in population 1 to be within the yellow basin. For $m = 0$, the required initial frequency for convergence to the DTE is 0.63; for higher migration rates, the initial frequency needs to be even higher. This means that considerable effort in the release phase is required to attain differential targeting.

Note that at the point that m^* is breached, moving from F to G in S14 Fig, the DTE (the yellow circle in S14F Fig) is found in the blue basin in S14G Fig, and therefore breaching of m^* results in global loss of the gene-drive allele. This is unlike the example in Fig 2, where breaching m^* results in global fixation.

References

1. Deredec A, Burt A, Godfray HCJ. The population genetics of using homing endonuclease genes in vector and pest management. Genetics. 2008;179(4):2013–2026.

2. Unckless RL, Messer PW, Connallon T, Clark AG. Modeling the manipulation of natural populations by the mutagenic chain reaction. Genetics. 2015;201(2):425–431.

3. Champer J, Chung J, Lee YL, Liu C, Yang E, Wen Z, et al. Molecular safeguarding of CRISPR gene drive experiments. eLife. 2019;8:1–10.

4. Grunwald HA, Gantz VM, Poplawski G, Xu XRS, Bier E, Cooper KL. Super-Mendelian inheritance mediated by CRISPR-Cas9 in the female mouse germline. Nature. 2019;566(7742):105–109.

5. Ewens W. Mathematical Population Genetics: I. Theoretical Introduction. 2nd ed. New York, NY: Springer; 2004.
