# Supplementary figures and images for: Designing gene drives to limit spillover to non-target populations
Source: PLoS Genet. 2021 Feb 25;17(2):e1009278. doi: 10.1371/journal.pgen.1009278 (PMC7943199; doi:10.1371/journal.pgen.1009278)

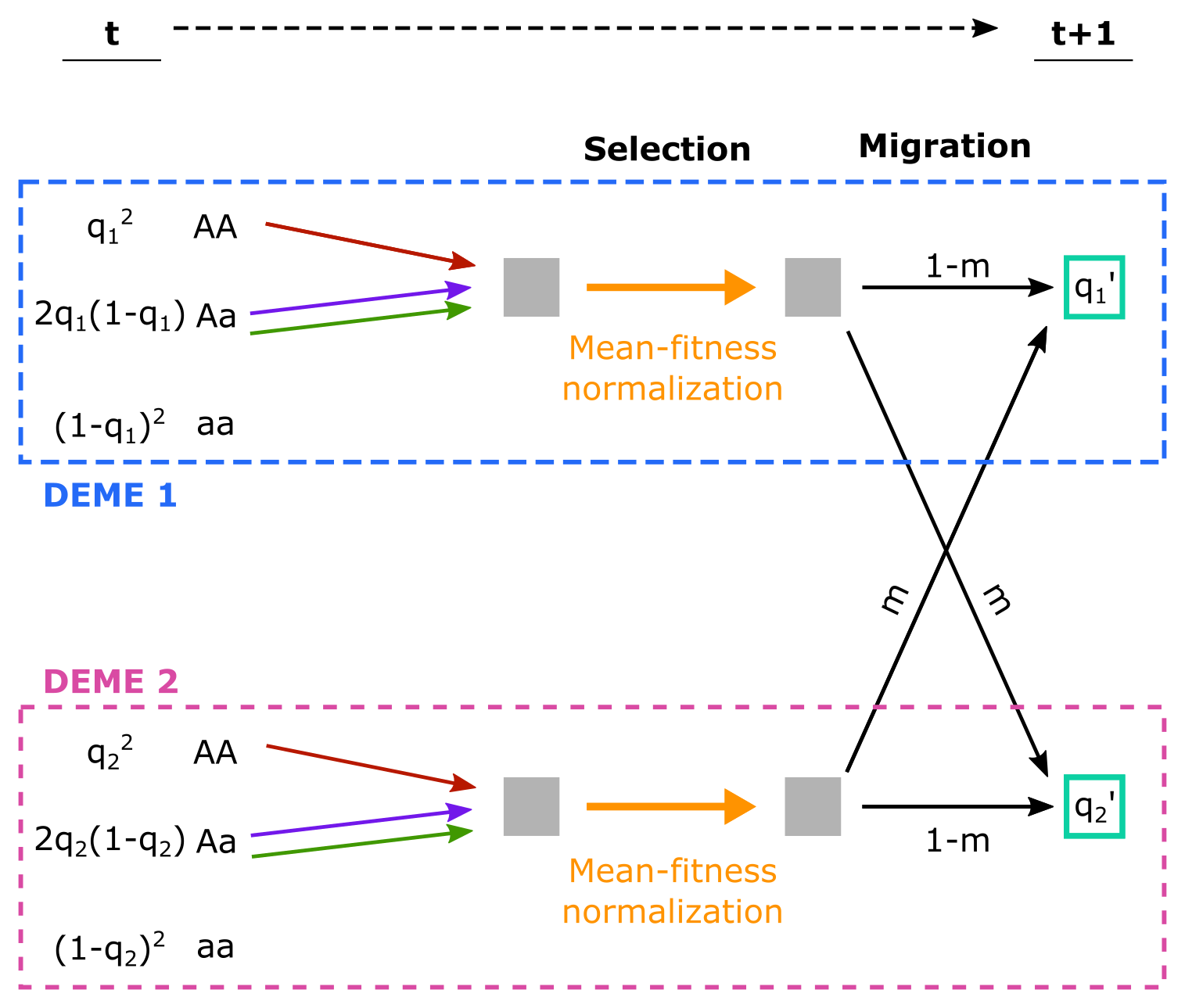

Supplement: S1 Fig — For interpretation of arrow colors, see Fig 1 in the main text. (PNG) [file pgen.1009278.s002.png]

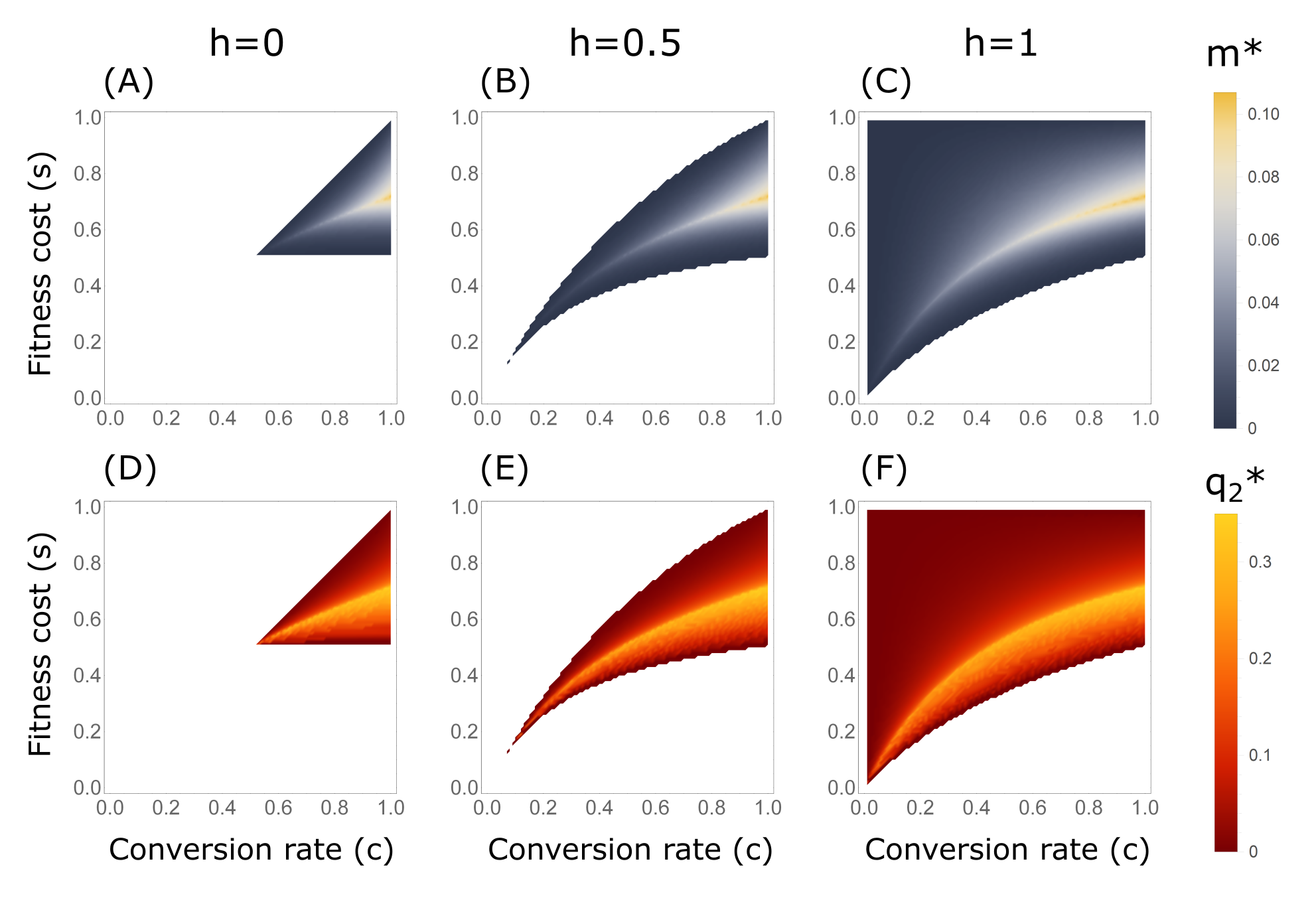

Supplement: S2 Fig — (A–C) Maximal migration rates m* for which a differential-targeting equilibrium (DTE) exists. The colored regions denote the configurations for which a DTE exists (B2 configurations), and the regions in white denote configurations for which differential targeting of the demes is not possible (A1, A2, and B1). Over most of the parameter space, a DTE exists only for low migration rates (in blue), and only in a narrow band do DTEs exist with migration rates above m* = 0.05 (light yellow). (D–F) Maximal gene-drive frequencies in the non-target population, q2*, at differential-targeting equilibria (DTEs). q2* values are correlated with m* values. The figure design follows Fig 3. (PNG) [file pgen.1009278.s003.png]

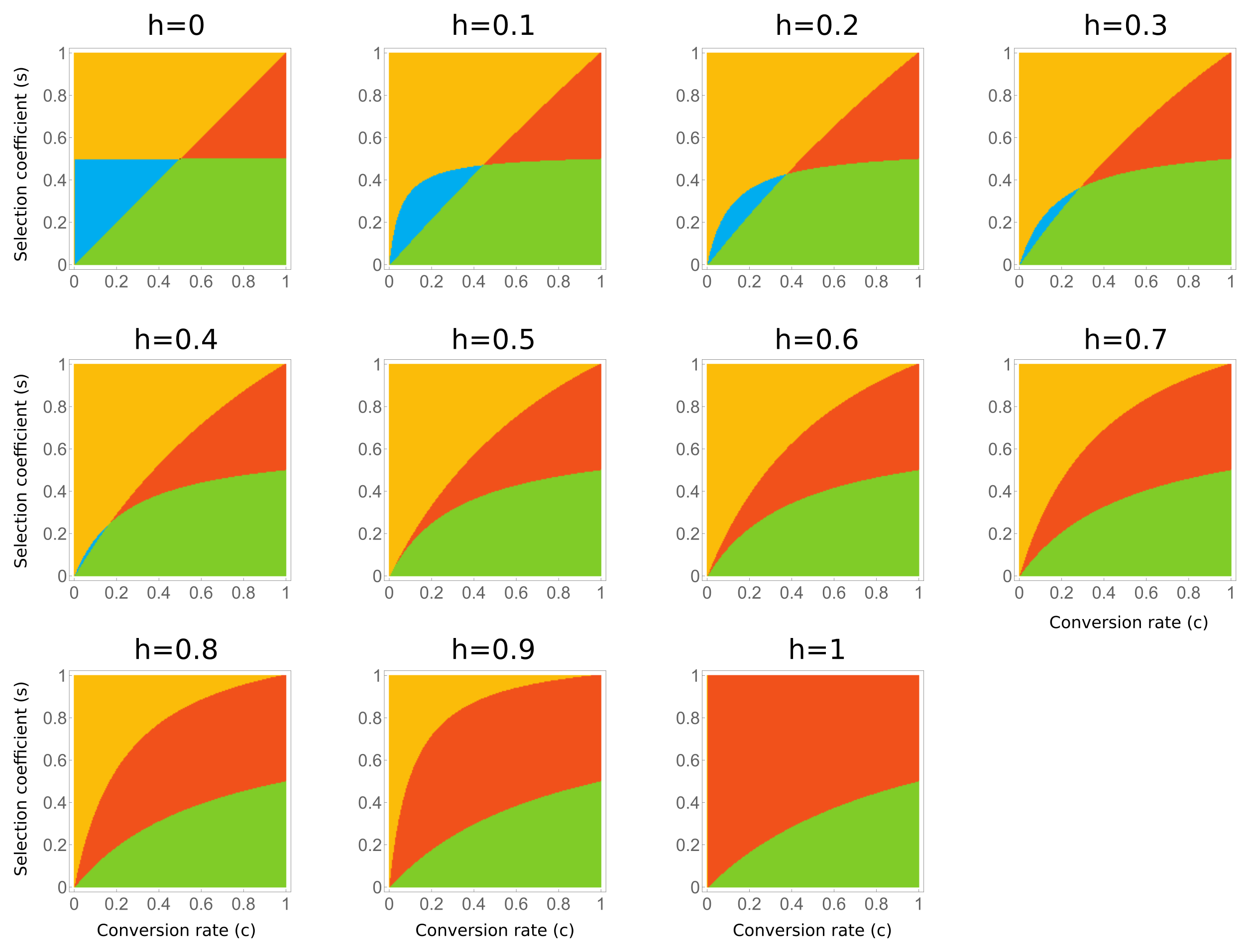

Supplement: S3 Fig — Green: A1 configurations, with two trivial equilibria, q^=0 (loss) unstable and q^=1 (fixation) stable; yellow: A2 configurations, with two trivial equilibria, q^=0 stable and q^=1 unstable; blue: B1 configurations, with 1 stable non-trivial equilibrium and 2 unstable trivial equilibria; red: B2 configurations, with 1 unstable non-trivial equilibrium and 2 stable trivial equilibria. The panels show results for 11 values of h and all possible values of s and c. (PNG) [file pgen.1009278.s004.png]

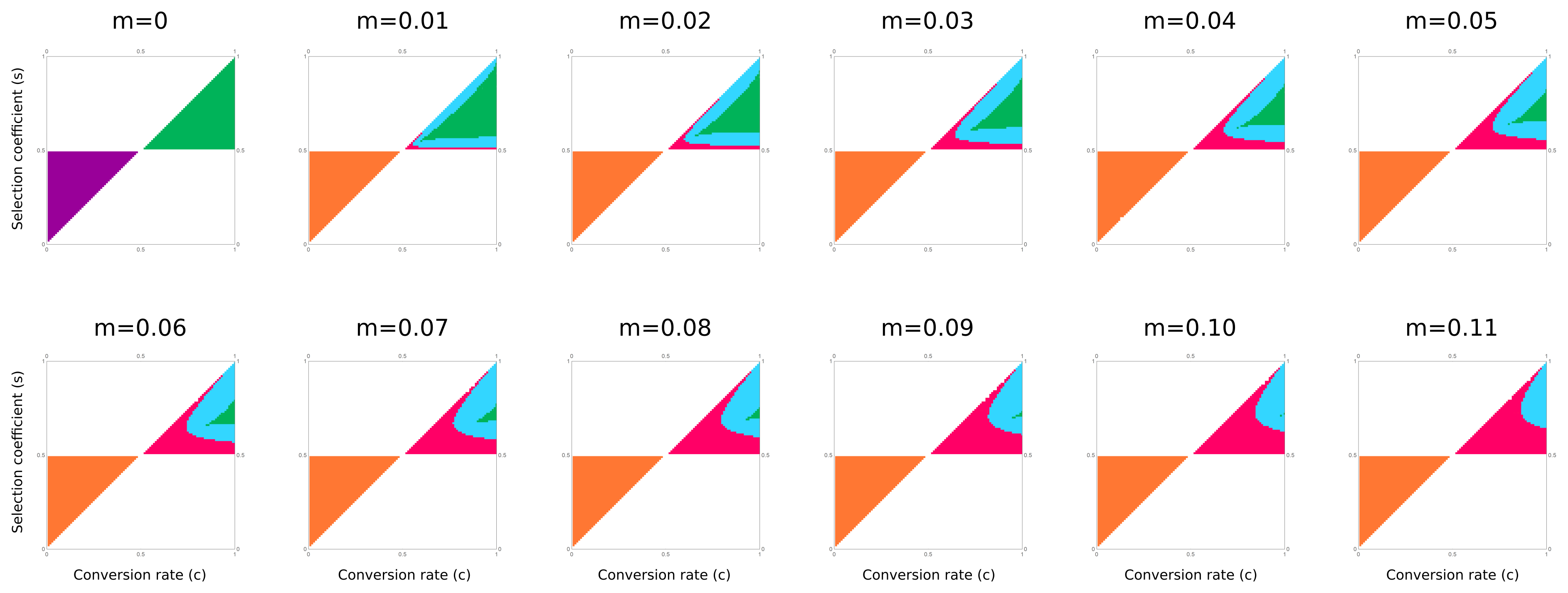

Supplement: S4 Fig — Green—9 equilibria, 4 stable and 5 unstable, one of which is a DTE (stable and q^1>q^2); purple—9 equilibria, 8 unstable and 1 stable and symmetric (q^1=q^2); blue—5 equilibria, 2 stable trivial (global fixation and global loss) and 3 unstable; red—3 equilibria, 2 stable trivial and 1 unstable; orange—3 equilibria, 2 unstable trivial and 1 stable symmetric; white—2 trivial equilibria, 1 stable and 1 unstable (A1 and A2 configurations). In the bottom white region, the stable equilibrium is global fixation of the gene-drive allele (A1 configuration), and in the top white region, the stable equilibrium is global loss (A2 configuration). The only gene-drive configurations for which differential targeting is possible appear in green. (PNG) [file pgen.1009278.s005.png]

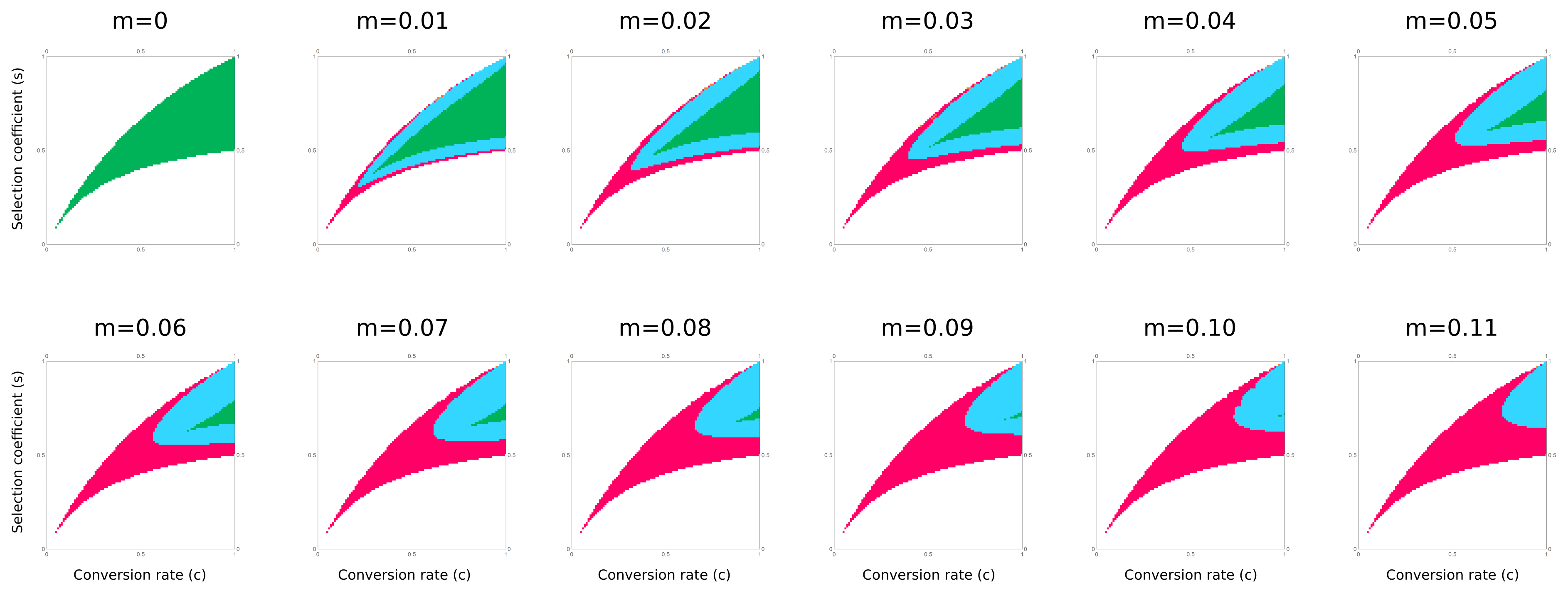

Supplement: S5 Fig — Green—9 equilibria, 4 stable and 5 unstable, one of which is a DTE (stable and q^1>q^2); purple—9 equilibria, 8 unstable and 1 stable and symmetric (q^1=q^2); blue—5 equilibria, 2 stable trivial (global fixation and global loss) and 3 unstable; red—3 equilibria, 2 stable trivial and 1 unstable; white—2 trivial equilibria, 1 stable and 1 unstable (A1 and A2 configurations). In the bottom white region, the stable equilibrium is global fixation of the gene-drive allele (A1 configuration), and in the top white region, the stable equilibrium is global loss (A2 configuration). The only gene-drive configurations for which differential targeting is possible appear in green. (PNG) [file pgen.1009278.s006.png]

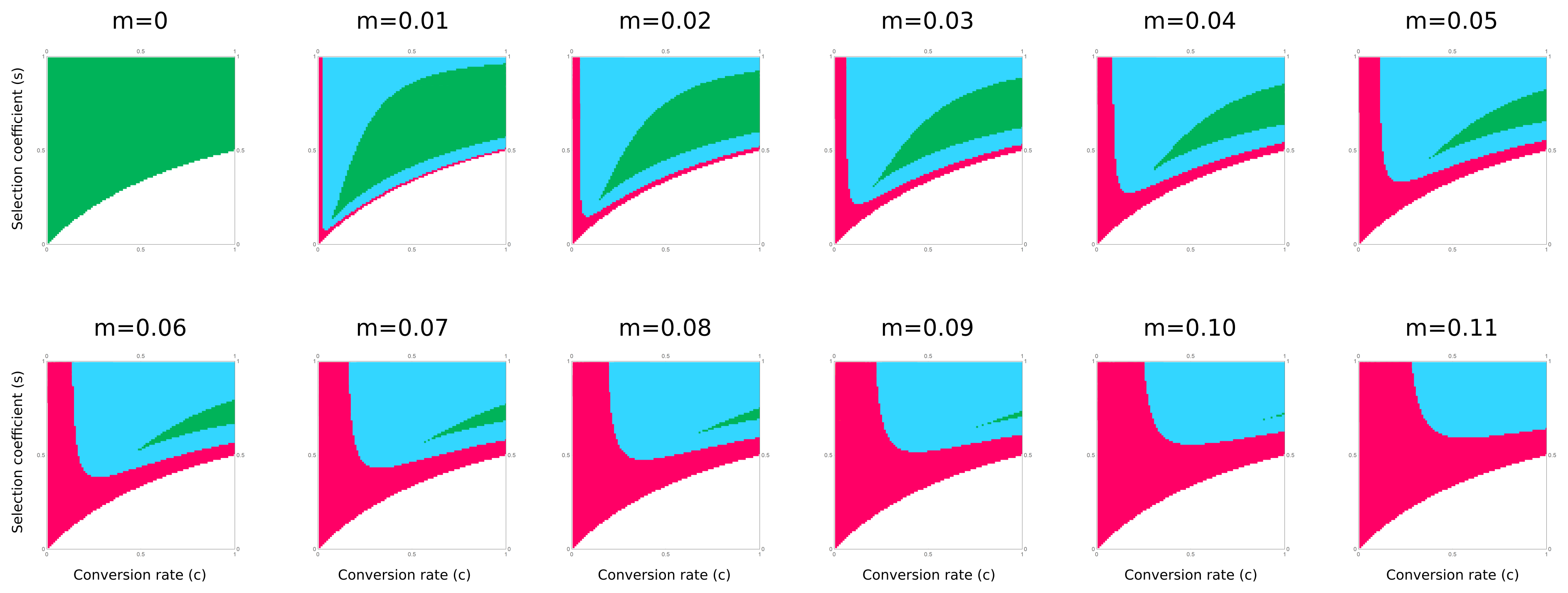

Supplement: S6 Fig — Green—9 equilibria, 4 stable and 5 unstable, one of which is a DTE (stable and q^1>q^2); purple—9 equilibria, 8 unstable and 1 stable and symmetric (q^1=q^2); blue—5 equilibria, 2 stable trivial (global fixation and global loss) and 3 unstable; red—3 equilibria, 2 stable trivial and 1 unstable; white—A1 configuration, with 1 stable (global fixation) and 1 unstable (global loss) equilibrium. The only gene-drive configurations for which differential targeting is possible appear in green. (PNG) [file pgen.1009278.s007.png]

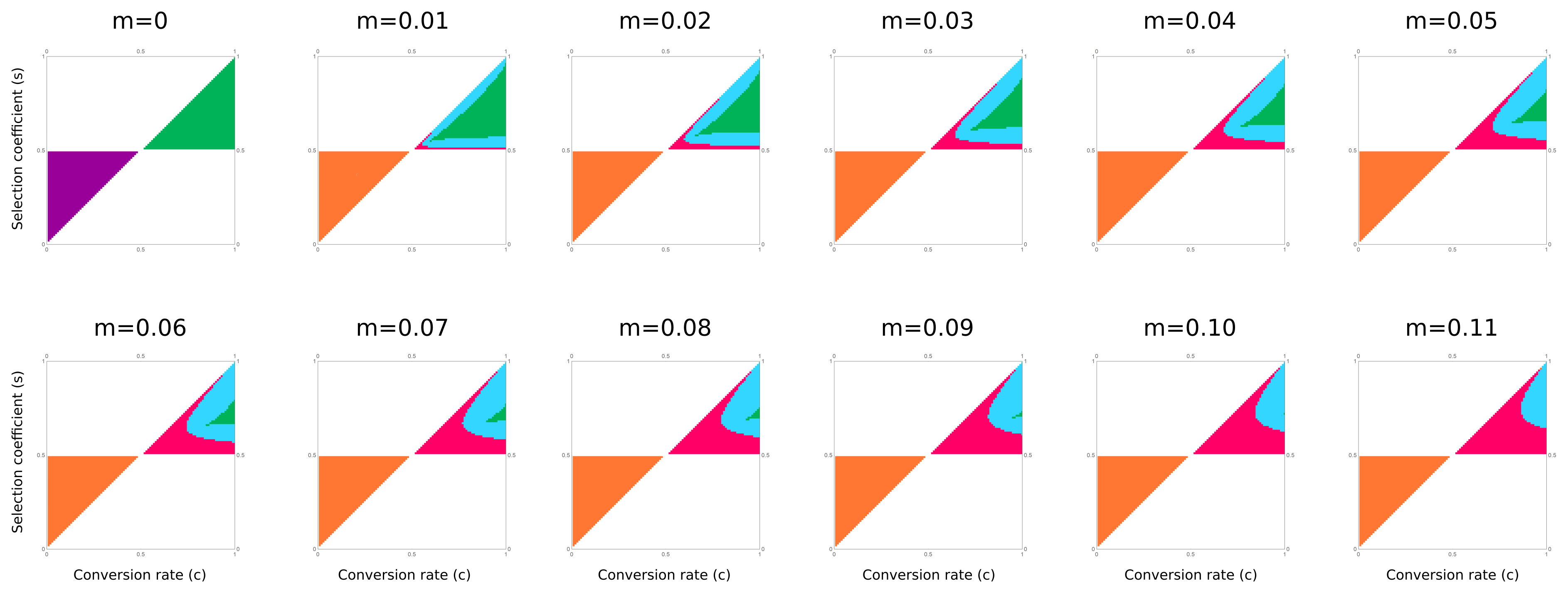

Supplement: S7 Fig — Green—9 equilibria, 4 stable and 5 unstable, one of which is a DTE (stable and q^1>q^2); purple—9 equilibria, 8 unstable and 1 stable and symmetric (q^1=q^2); blue—5 equilibria, 2 stable trivial (global fixation and global loss) and 3 unstable; red—3 equilibria, 2 stable trivial and 1 unstable; orange—3 equilibria, 2 unstable trivial and 1 stable symmetric; white—2 trivial equilibria, 1 stable and 1 unstable (A1 and A2 configurations). In the bottom white region, the stable equilibrium is global fixation of the gene-drive allele (A1 configuration), and in the top white region, the stable equilibrium is global loss (A2 configuration). The only gene-drive configurations for which differential targeting is possible appear in green. (PNG) [file pgen.1009278.s008.png]

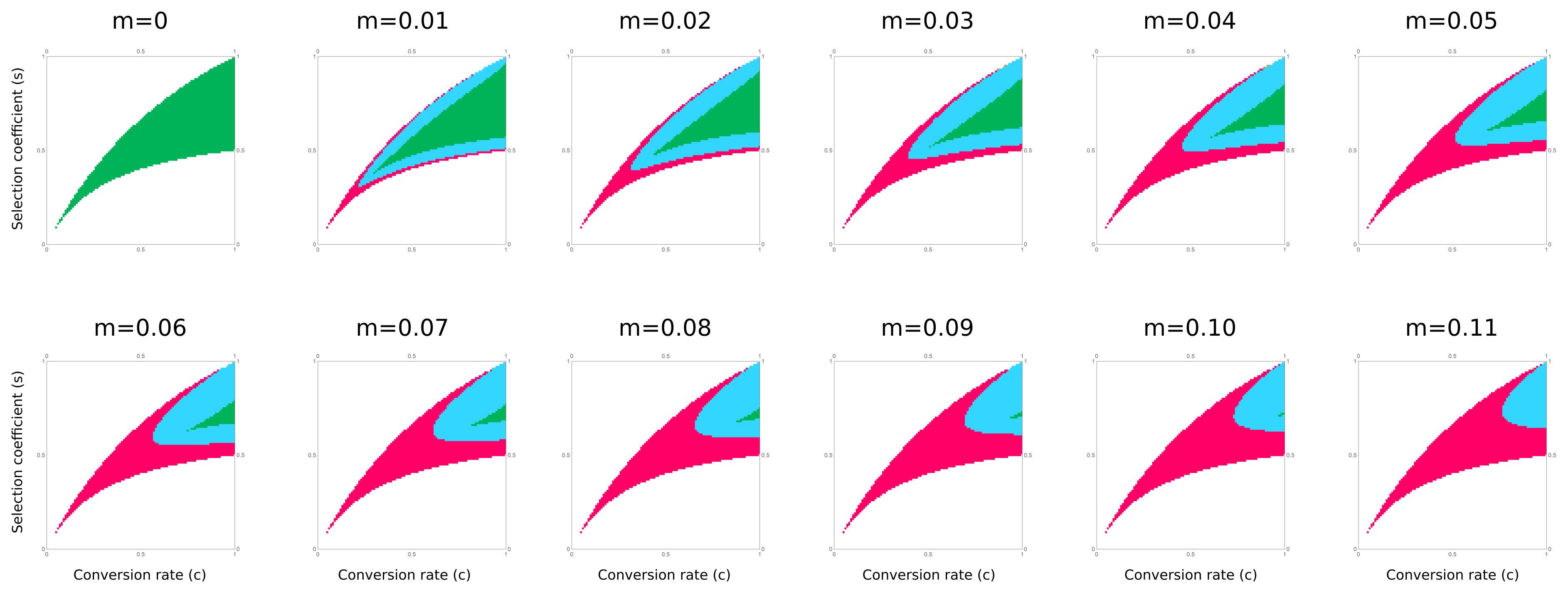

Supplement: S8 Fig — Green—9 equilibria, 4 stable and 5 unstable, one of which is a DTE (stable and q^1>q^2); purple—9 equilibria, 8 unstable and 1 stable and symmetric (q^1=q^2); blue—5 equilibria, 2 stable trivial (global fixation and global loss) and 3 unstable; red—3 equilibria, 2 stable trivial and 1 unstable; white—2 trivial equilibria, 1 stable and 1 unstable (A1 and A2 configurations). In the bottom white region, the stable equilibrium is global fixation of the gene-drive allele (A1 configuration), and in the top white region, the stable equilibrium is global loss (A2 configuration). The only gene-drive configurations for which differential targeting is possible appear in green. (PNG) [file pgen.1009278.s009.png]

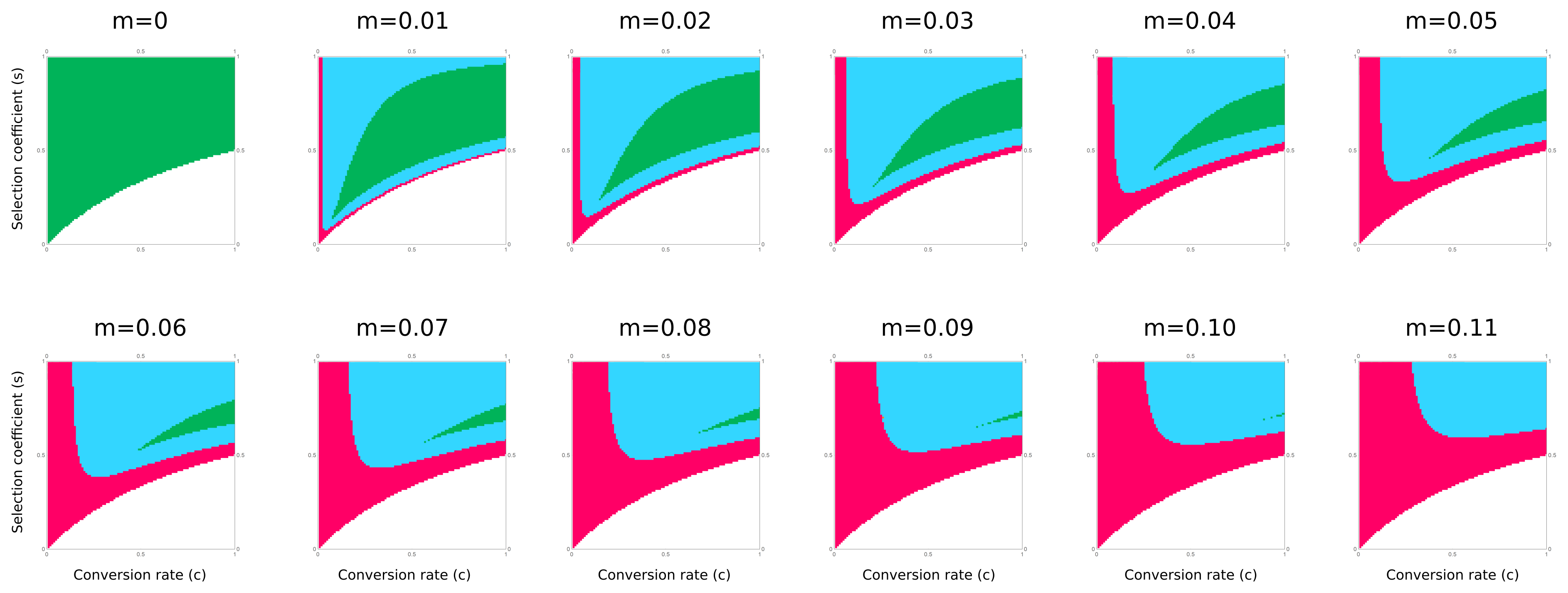

Supplement: S9 Fig — Green—9 equilibria, 4 stable and 5 unstable, one of which is a DTE (stable and q^1>q^2); purple—9 equilibria, 8 unstable and 1 stable and symmetric (q^1=q^2); blue—5 equilibria, 2 stable trivial (global fixation and global loss) and 3 unstable; red—3 equilibria, 2 stable trivial and 1 unstable; white—A1 configuration, with 1 stable (global fixation) and 1 unstable (global loss) equilibrium. The only gene-drive configurations for which differential targeting is possible appear in green. (PNG) [file pgen.1009278.s010.png]

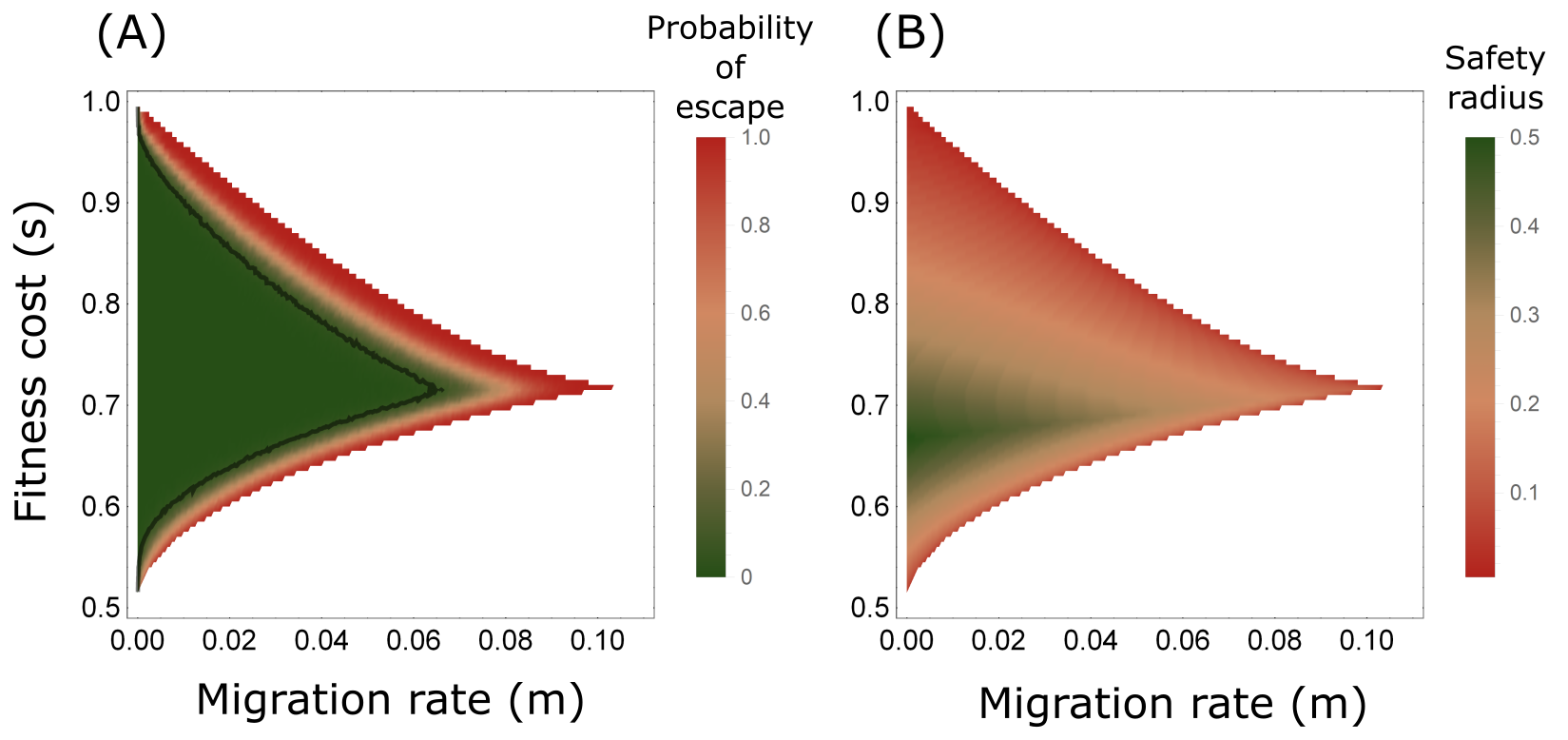

Supplement: S10 Fig — Results shown for gene drives with full conversion, c = 1, and for different migration rates m and selection coefficients s. White regions denote scenarios for which m > m*. (A) The probability of escape from the DTE due to genetic drift, defined as the probability of departing from the attraction basin of the DTE over 100 generations with genetic drift, in a Wright-Fisher population with Ne = 100. The black line denotes 5% probability of escape. Probabilities were estimated from 1000 simulated replicates. (B) The safety radius of the DTE for the m-before-s model. (PNG) [file pgen.1009278.s011.png]

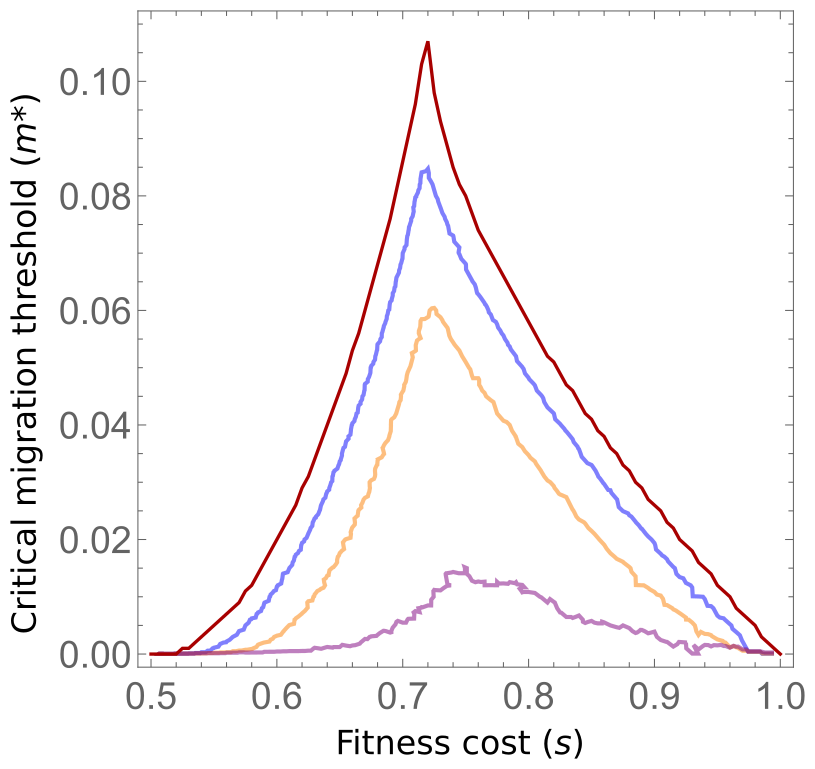

Supplement: S11 Fig — The critical migration threshold m* for the two-deme model in Eq 3 with c = 1 is shown in red. Other curves show the threshold for probability of escape >0.05% for different effective population sizes of the demes, for the same model, computed with 1000 simulated iterations of Eq S3 for each gene-drive configuration (equivalent to black line in Fig 5A and S10A Fig). Ne = 200 in blue; Ne = 50 in yellow; Ne = 10 in purple. Critical migration thresholds are effectively lower with lower effective population sizes. (PNG) [file pgen.1009278.s012.png]

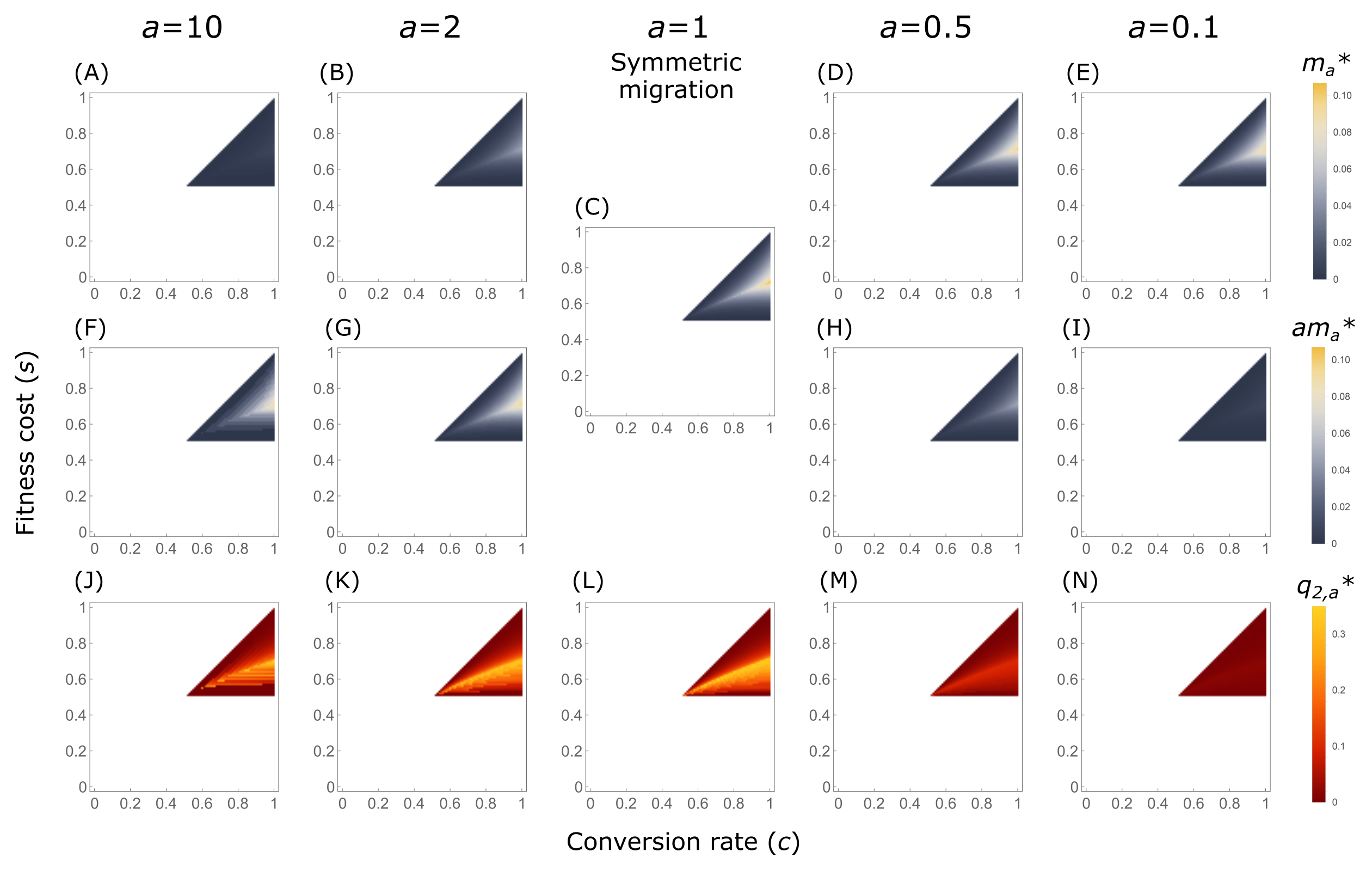

Supplement: S12 Fig — (A–E) Critical migration thresholds from the non-target deme to the target deme, ma*, for migration ratio a. (F–I) Critical migration thresholds expressed as migration from target to non-target demes, ama*, for migration ratio a. (J–N) Maximal gene-drive frequencies in the non-target population at the DTE, q2,a*, with migration ratio a. (PNG) [file pgen.1009278.s013.png]

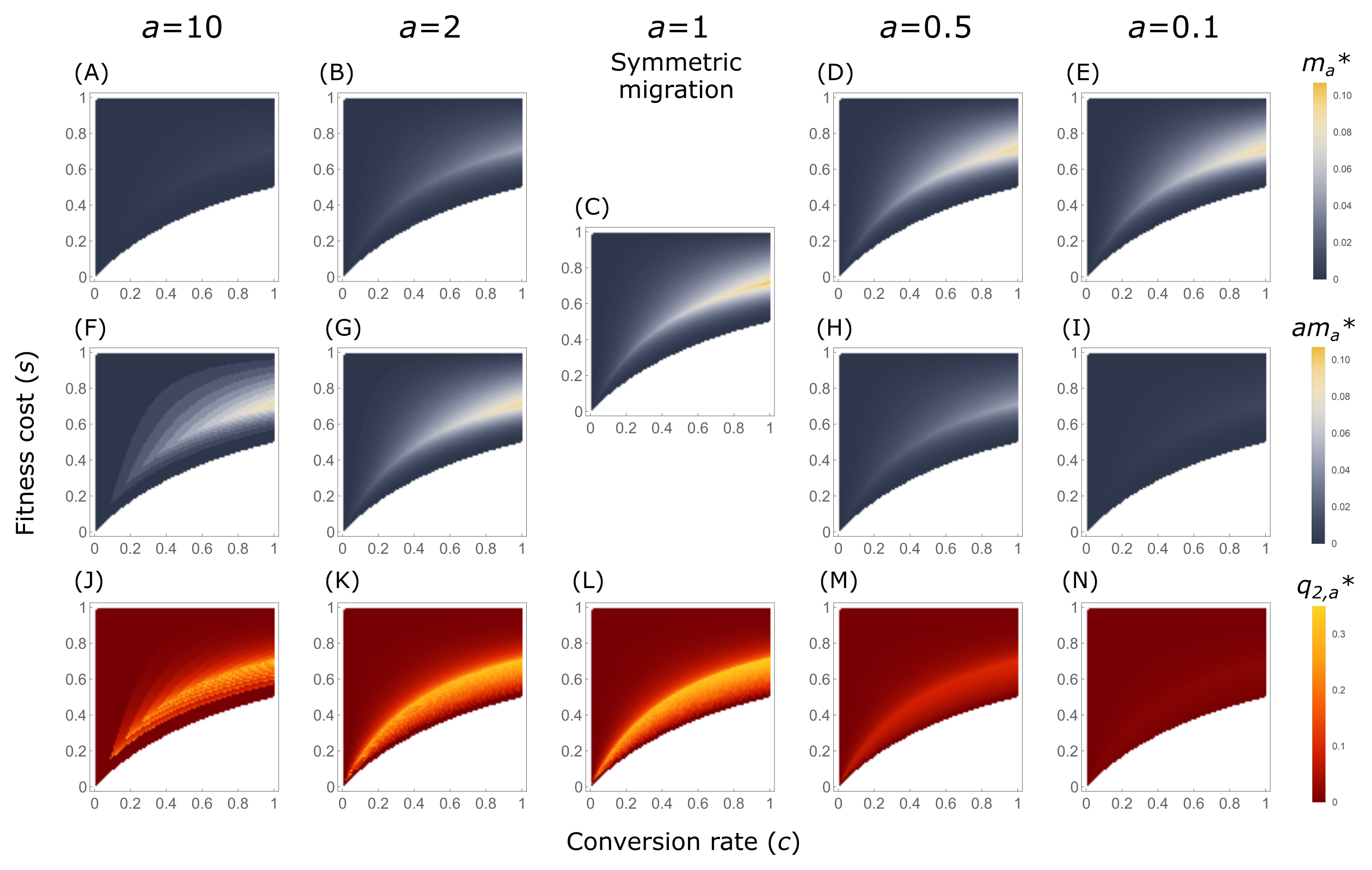

Supplement: S13 Fig — (A–E) Critical migration thresholds from the non-target deme to the target deme, ma*, for migration ratio a. (F–I) Critical migration thresholds expressed as migration from target to non-target demes, ama*, for migration ratio a. (J–N) Maximal gene-drive frequencies in the non-target population at the at DTE, q2,a*, with migration ratio a. (PNG) [file pgen.1009278.s014.png]

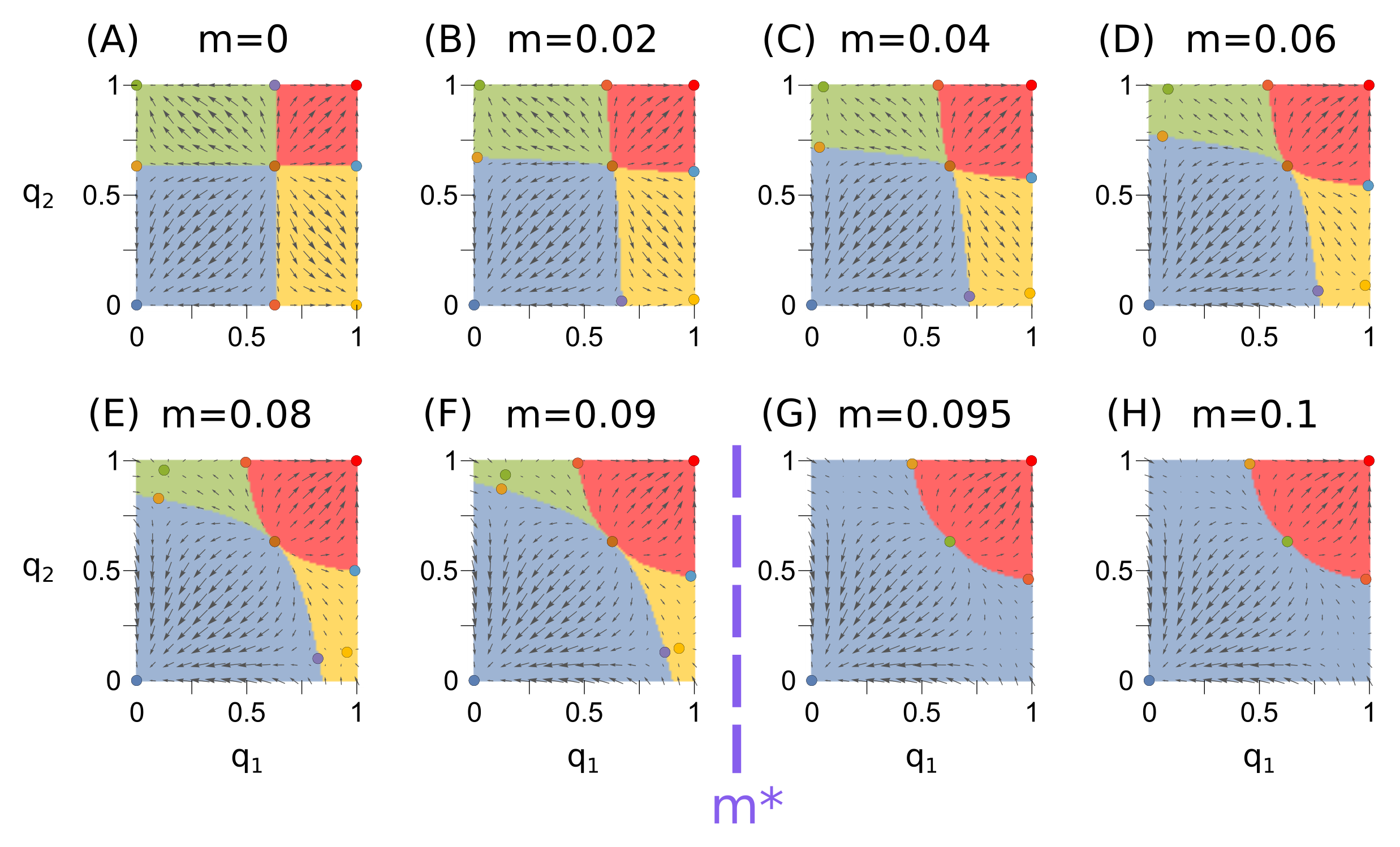

Supplement: S14 Fig — Shown are results for the m-before-s model with a gene-drive configuration (s, c, h) of c = 1, s = 0.73 and arbitrary h, as per the malaria-vector example in the main text. The circles show the equilibria. The colored regions show the attraction basins, with the basin colors corresponding to the stable equilibria. The arrows show the vector field that describes the magnitude and direction of the change in allele frequencies at each point in frequency space. The differential-targeting equilibrium (DTE) is the stable yellow equilibrium point, which exists for migration rates lower than m* ≈ 0.093. (PNG) [file pgen.1009278.s015.png]
